# The Phenomenon of Antiretroviral Drug Resistance in the Context of Human Immunodeficiency Virus Treatment: Dynamic and Ever Evolving Subject Matter

**DOI:** 10.3390/biomedicines12040915

**Published:** 2024-04-20

**Authors:** Miruna-Maria Apetroaei, Bruno Ștefan Velescu, Marina Ionela (Ilie) Nedea, Cristina Elena Dinu-Pîrvu, Doina Drăgănescu, Anca Ionela Fâcă, Denisa Ioana Udeanu, Andreea Letiția Arsene

**Affiliations:** 1 Faculty of Pharmacy, Carol Davila University of Medicine and Pharmacy, 6 Traian Vuia Street, 020956 Bucharest, Romania; miruna-maria.apetroaei@rez.umfcd.ro (M.-M.A.); marina.nedea@umfcd.ro (M.I.N.); cristina.dinu@umfcd.ro (C.E.D.-P.); doina.draganescu@umfcd.ro (D.D.); anca-ionela.faca@drd.umfcd.ro (A.I.F.); denisa.udeanu@umfcd.ro (D.I.U.); andreea.arsene@umfcd.ro (A.L.A.); 2 Marius Nasta Institute of Pneumophthisiology, 90 Viilor Street, 050159 Bucharest, Romania

**Keywords:** antiretroviral drug resistance, ART drug resistance, HIV genetic variability, ART advancements, global HIV/AIDS management

## Abstract

Human immunodeficiency virus (HIV) is a significant global health issue that affects a substantial number of individuals across the globe, with a total of 39 million individuals living with HIV/AIDS. ART has resulted in a reduction in HIV-related mortality. Nevertheless, the issue of medication resistance is a significant obstacle in the management of HIV/AIDS. The unique genetic composition of HIV enables it to undergo rapid mutations and adapt, leading to the emergence of drug-resistant forms. The development of drug resistance can be attributed to various circumstances, including noncompliance with treatment regimens, insufficient dosage, interactions between drugs, viral mutations, preexposure prophylactics, and transmission from mother to child. It is therefore essential to comprehend the molecular components of HIV and the mechanisms of antiretroviral medications to devise efficacious treatment options for HIV/AIDS.

## 1. Introduction

Since its discovery in the early 1980s, human immunodeficiency virus (HIV) has emerged as a significant public health concern affecting the lives of millions of individuals. The mortality resulting from HIV infection and the emergence of acquired immune deficiency syndrome (AIDS) has imposed a significant burden on global healthcare systems. The World Health Organization (WHO) estimated that there were 38.4 million individuals living with HIV in 2021, with 1.5 million having tested positive for the virus in just one year alone [1].

HIV belongs to the subfamily *Orthoretrovirinae* of the family *Retroviridae* in the genus *Lentivirus* and is categorized into two types, HIV-1 and HIV-2, based on genetic features and variations in viral antigens [2,3].

HIV-1 and HIV-2 exhibit numerous similarities, encompassing fundamental gene arrangement, routes of transmission, intracellular replication pathways, and clinical outcomes, culminating in the development of AIDS [4,5]. HIV-2 is distinguished by diminished transmission and a decreased probability of advancing to AIDS [4,6]. From an epidemiological perspective, HIV-2 is predominantly restricted to West Africa, while HIV-1 has a global distribution. From a clinical perspective, it appears that individuals infected with HIV-2 can be divided into two distinct groups, with the majority exhibiting long-term non-progression, while the majority of those infected with HIV-1 tend to experience disease progression. In instances of clinical evolution, both viruses exhibit comparable pathological mechanisms, albeit with HIV-2 manifesting progression at elevated CD4 counts. HIV-2 infection is characterized by consistently lower plasma viral loads and fewer typical instances of immune activation [4,7,8].

The implementation of antiretroviral therapy (ART) has significantly transformed the landscape of HIV/AIDS care, resulting in a substantial reduction in mortality rates and disease severity [9], as well as a decrease in mother-to-child transmission [10,11,12]. However, the success rate of ART is jeopardized by the development of resistance to antiretroviral drugs. The development of strategies to avoid or overcome medication resistance requires an understanding of the cellular and molecular underpinnings of the HIV virus, as well as the underlying mechanisms of therapeutic action. This article aims to discuss the importance of this knowledge in HIV/AIDS management and present the latest findings in this field of study. Our goal is to thoroughly analyze the safety, effectiveness, and constraints of the existing pharmacological treatments utilized in the management of HIV infection.

## 2. HIV Structure and Genome

**Figure 1:**
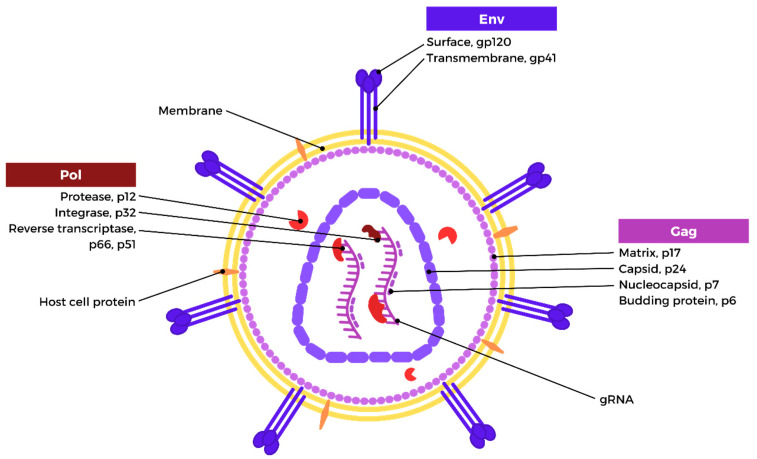
HIV-1 structure (created with Biorender.com).

The mature HIV-1 viral particles are spherical, with a diameter of approximately 100 nm, and are surrounded by an envelope [13,14]. This envelope of the viral particle consists of a bilayer of lipids that originates from the host cell membrane after the process of immuration, whereby the viral particle detaches from the host cell. The envelope is composed of a combination of viral and host cell proteins [15,16]. Viral envelope proteins (Env) are glycoprotein heterotrimers that are comprised of surface and transmembrane subunits that are not covalently linked. The gp41 transmembrane subunit is responsible for the anchoring of gp120 and the fusion of the viral and cellular membranes, whereas the gp120 surface subunit is essential for enabling the virus to attach to the target cell [16,17]. Under the virion membrane, and surrounding the capsid core, is a myristoylated layer of matrix protein (p17), which forms the inner membrane layer. The capsid core is generated by the capsid protein (p24), which condenses into a truncated cone following virion maturation. The capsid core includes two copies of positive single-strand genomic RNA bound to the nucleocapsid (p7) and the reverse transcriptase (p51) and integrase (p32) [14,17]. The formation of the nucleoprotein/RNA complex is attributed to the nucleocapsid. Reverse transcriptase is accountable for the conversion of HIV RNA into proviral DNA, while the integration of proviral DNA into the host genome is facilitated by the integrase enzyme. The protease enzyme (p10) situated in the interstitial space between the matrix and capsid structures of the virus is primarily responsible for executing the proteolytic cleavage of the precursor proteins Gag (Pr55) and Gag-Pol (Pr160GagPol), leading to the liberation of both the structural proteins and viral enzymes [15,16,17]. The HIV structure is depicted in Figure 1.

The HIV genome consists of several key structural and regulatory genes, with the former comprising *gag*, *pol*, and *env* and the latter including *tat*, *rev*, *nef*, *vif*, *vpr*, and *vpu*. These genes are pivotal to the viral replication cycle and contribute to the pathogenic effects of HIV [17,18,19]. Additionally, long terminal repeat (LTR) sequences, which are present at both ends of the proviral DNA, facilitate the integration of the viral genetic material into the host cell genome. The 5′ LTR segment encodes the promoter for viral transcription of genes [17,20]. The genetic composition of HIV comprises several essential genes that perform a pivotal function in the virus’s replication process. The *gag* gene is accountable for the synthesis of three crucial proteins, namely p24, p6, and p7, which function as integral constituents of the viral capsid, nucleocapsid, and matrix protein. The *pol* gene is responsible for encoding the reverse transcriptase, integrase, and protease enzymes. These enzymes perform the crucial functions of converting the viral RNA into double-stranded DNA, incorporating the DNA into the host’s genome, and converting viral proteins into operational proteins, respectively. Additionally, it can be noted that the *env* gene plays a crucial role in the encoding of gp160. This protein is subsequently transformed into the gp120 and gp41 proteins, which are integral components of the viral envelope spikes. These spikes are responsible for facilitating the attachment of the virus and fusion with host cells [17,21,22,23].

**Figure 2:**
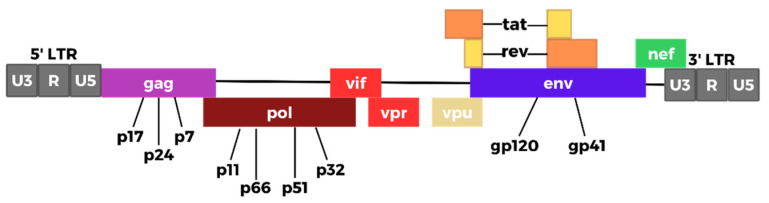
HIV genome (adapted from [19]) (created with Biorender.com).

Trans-activator of transcription (*Tat*) acts as a messenger RNA elongator and trans-activator, promoting efficient viral replication. It also induces apoptosis, which helps the virus evade immune surveillance [24,25]. Regulator of virion (*Rev*) regulates the transport and processing of viral mRNA, while negative factor (*Nef*) increases retrotranscription and negatively regulates CD4 and HLA class I, thereby promoting viral replication and further immune evasion. Viral infectivity factor (*Vif*) increases viral infectivity and interacts with APOBEC3G, which normally limits the replication of HIV. Viral protein R (*Vpr*) is a viral trans-activator that helps transport the pre-integration complex to the nucleus, inducing cell cycle arrest and apoptosis, and therefore helping the virus evade immune responses. Viral protein U (*Vpu*) sequesters CD4 in the endoplasmic reticulum, further suppressing immune responses and increasing the generation and release of virions, thereby promoting viral spread [26,27,28,29,30]. Figure 2 depicts the HIV genome.

## 3. HIV Therapeutic Targets and Current Pharmacological Approaches

During the earliest days of the HIV epidemic, a positive HIV diagnosis was accompanied by an unfavorable prognosis and limited treatment options for infected individuals. With the development of antiretroviral therapy, the prognosis for HIV-positive individuals has risen significantly [31,32]. Modern antiretroviral therapy has transformed HIV from a fatal disease into a chronic condition, allowing infected individuals to live an almost normal life [33,34,35].

Multiple classes of drugs, such as nucleoside and non-nucleoside reverse transcriptase inhibitors, integrase strand transfer inhibitors, protease inhibitors, and entry inhibitors have demonstrated high efficacy in suppressing viral replication and restoring immune function in HIV-positive patients. These drug classes are recommended by the WHO consolidated guidelines published in July 2021 [36].

From 1983–1984, the AIDS causative agent was identified and characterized, and in 1984, the very first reverse transcriptase inhibitor, zidovudine, became available. Over the next decade, further inhibitors from the same class were developed, resulting in the introduction of highly active antiretroviral therapy in 1996, which significantly enhanced patient survival. In the years that followed, additional categories of drugs were introduced, including non-nucleoside inhibitors in 1997, viral entry inhibitors in 2002, and integration inhibitors and CCR5 coreceptors in 2007. The implementation of these novel treatment regimens had a profound impact on the management and long-term prognosis of patients who have not previously responded to antiretroviral medication [26,37].

Antiretroviral treatment is directed at the three enzymes encoded by HIV-1, including reverse transcriptase (RT), protease (PR), and integrase (IN). During the maturation phase of the HIV life cycle, PR is synthesized as a component of the Gag-Pol precursor polyprotein, which is encoded in the viral genome [38,39,40]. PR is accountable for transforming Gag and Gag-Pol precursors into mature viral proteins. The identification of the constant catalytic residues Asp-Thr/Ser-Gly established PR as an identifiable member of the aspartic protease family [40]. In infected cells, the human immunodeficiency virus type 1 reverse transcriptase enzyme catalyzes the reverse transcription of the virus’s RNA genome into double-stranded DNA. This is an essential early phase in the maturation cycle of the virus. The *pol* open frame of reference encodes RT, which is translated into a Gag-Pol protein precursor and then proteolyzed by viral protease (PR) into 66 kDa (p66) and 51 kDa (p51) subunits, with active RT produced as a heterodimer of p66 and p51 [41,42]. IN is responsible for two processes, both of which involve the same active site in the molecule. A (GT)n dinucleotide is eliminated from both 3′ extremities of the linear viral DNA that is generated by RT in the first step, known as 3′-processing (3′-P) [43,44]. Each of the 3′-ends of the viral DNA undergoes processing, leaving a preserved (CA) dinucleotide. The hydroxyl groups found in the deoxyadenosine nucleotides at the 3′ ends of the transformed viral DNA act as nucleophiles to attack the host genome, resulting in a substitution reaction in which the 3′ ends of the viral DNA are incorporated into the host DNA [45,46]. IN catalyzes this process, which is called the strand transfer (ST) reaction. Due to the configuration of the transfer event, the host sequences that border the integrated viral DNA undergo a brief duplication [47,48]. Host DNA repair enzymes correct an inconsistency left by the integration process [49,50], and the result is a provirus, which is a firmly incorporated form of viral DNA.

**Figure 3:**
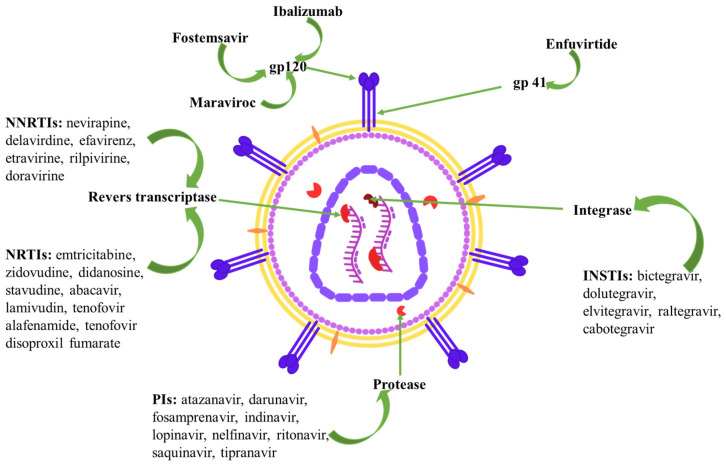
Drug classes used to treat HIV infection. Legend: NRTI—nucleoside reverse transcriptase inhibitors; NNRTI—non-nucleoside reverse transcriptase inhibitors; INSTI—integrase strand transfer inhibitors; PI—protease inhibitors.

The European Society for AIDS has recently revised its recommendations for antiretroviral therapy, which involves the administration of several different drugs. Figure 3 depicts the sites of action of the various drug classes used to treat HIV infection.

Despite the fact that a number of therapeutic options have been shown to have comparable levels of efficacy, there are differences in dosage frequency, the number of tablets required, interactions between drugs, and potential adverse effects. The selection of a specific regimen for an individual is predominantly influenced by factors such as anticipated adverse effects, functionality, the presence of other medical conditions, the possibility of drug interactions, and the findings of genotypic drug resistance testing [51]. Table 1 summarizes the classes of HIV infection-treating drugs, their mechanisms of action, and adverse reactions.

The class of nucleoside reverse transcriptase inhibitors (NRTIs) contains the following molecules: emtricitabine (FTC), zidovudine (ZDV), didanosine (ddI), stavudine (d4T), abacavir (ABC), lamivudine (3TC), tenofovir alafenamide (TAF), and tenofovir disoproxil fumarate (TDF) [59]. The HIV-1 reverse transcriptase enzyme is inhibited by NRTIs, which compete with natural nucleosides (such as dTTP, dCTP, dGTP, and dATP) and perform their function by incorporation into viral DNA. NRTIs are prodrugs that must undergo intracellular anabolic phosphorylation prior to being metabolized into their biologically active form as phosphorylated NRTI metabolites [26,60]. Upon entering the host cell, the drug is activated by cellular kinases, subsequently exerting its effect through its structural composition. Until the substance undergoes conversion to its active di- or triphosphate metabolite structure, NRTIs are inactive. Nonetheless, the distribution and metabolism of NRTIs are heavily dependent on the concentrations of functional NRTI derivatives and endogenous dNTP [61,62]. NRTIs possess a nucleoside or nucleotide as a base and lack a 3′-hydroxyl group at the 2′-deoxyribosyl moiety, which makes it possible for the NRTIs to inhibit the generation of a 3′-5′-phosphodiester bond in developing DNA chains, thereby inhibiting viral replication. The insertion of these drugs through RNA-dependent DNA or DNA-dependent DNA synthesis suppresses the generation of both positive and negative strands of DNA [59,63]. In summary, they perform through two mechanisms, as follows: by competing with natural nucleotides for insertion into the nascent DNA strand that is produced by reverse transcriptase, as well as “chain terminators” in viral genomic DNA assembly. For fundamentally historical reasons, ART in HIV-positive patients is built around a combination of two drugs from this class plus an NNRTI or PI because it has shown excellent effectiveness in limiting viral replication [26,61].

Non-nucleoside reverse transcriptase inhibitors (NNRTIs) are a class of antiretroviral drugs that have played a significant role in the treatment of human immunodeficiency infection. The first-generation NNRTIs, such as nevirapine (NVP), delavirdine (DLV), and efavirenz (EFV), have shown limited efficacy due to their low genetic barrier and poor resistance profile. To overcome these limitations, second-generation NNRTIs, including etravirine (ETR), rilpivirine (RPV), and doravirine (DOR), have been developed. The development of second-generation NNRTIs has significantly improved the therapeutic options available for HIV-infected patients, and the use of these drugs in combination with other antiretroviral drugs has resulted in better treatment outcomes [51]. NNRTIs are an important class of antiretroviral drugs used in combination therapy for HIV infection due to their high specificity, low toxicity, and unique antiviral activity [64,65,66]. NNRTIs effectively block the polymerization activity of HIV reverse transcriptase, which is responsible for synthesizing double-stranded viral DNA genomes from a single-stranded viral RNA genome [67]. By targeting the reverse transcriptase enzyme, they bind to an allosteric site called the NNRTI binding pocket (NNIBP), which is about 10 Å away from the DNA polymerase active site. Despite having different chemical structures, NNRTIs share a similar binding mode in which they adopt a “butterfly”, “horseshoe”, or “U” conformation with a central scaffold and two “wings” [68,69,70].

Antiretroviral protease inhibitors (PI) operate as pharmaceutical agents that impede the process of viral polyprotein precursor cleavage, hence impeding the production of functional proteins necessary for viral replication. These drugs are created via rational drug design, which relies on molecules that interact by binding to the catalytic site of the HIV protease. These diverse compounds, frequently exhibiting peptide-like characteristics, bear resemblance to the brief peptide that is broken by the viral protease, albeit with minimal structural resemblance. Protease structures, which are defined in three dimensions, are commonly obtained through X-ray crystallographic investigations [71]. The current available PIs encompass saquinavir, indinavir, ritonavir, nelfinavir, lopinavir, amprenavir, fosamprenavir, atazanvir, tipranavir, and darunavir [72].

The infection caused by HIV-1 relies on the incorporation of viral DNA into the chromatin of the host. The process of integration is facilitated by the viral enzyme integrase and encounters inhibition through the use of integrase strand transfer inhibitors (INSTIs) [73]. There are five INSTIs that have been approved for use in HIV patients, including bictegravir (BIC), dolutegravir (DTG), elvitegravir (EVG), raltegravir (RAL), and cabotegravir (CAB). Given that the HIV integrase catalyzes the irreversible incorporation of HIV reverse-transcribed viral DNA into the host genome via a pair of successive reactions known as 3′ processing and strand transfer [74], INSTIs are specifically targeting the subsequent step of the integration process [48,75] by coordinating Mg^2+^ and Mn^2+^ ions and Pi-stacking with the long terminal repeats that are found at each terminus of reverse-transcribed DNA fragments. D64, D116, and E152 (DDE) form a catalytic trio necessary for the coordination of divalent ions. INSTIs target the strand transfer phase of integration by binding to integrase [48,75].

For HIV-1 to infect CD4+ T cells, it must first identify and attach to the CD4 receptor that is located on the cell surface of the target cell. After that, it has to bind to either CCR5 or CXCR4, which are the two probable chemokine coreceptors. Before considering treatment options, it is necessary to determine whether the virus has an R5, X4, or dual/mixed (able to utilize both) tropism. R5 viruses use CCR5, whereas X4 viruses use CXCR4. Maraviroc (MVC) has been described as an entry inhibitor for HIV-1 treatment. By changing the structure of C-chemokine receptor 5 (CCR5), MVC blocks the virus’s ability to adhere to and enter CD4+ T-lymphocytes. MVC attaches to the CCR5 receptor at a different position than HIV-1 and produces a conformational shift in the receptor, rendering it unidentifiable to the virus. Consequently, MVC inhibits HIV-1’s capacity to complete the second phase in the viral entry process, chemokine coreceptor binding, in a noncompetitive manner. This restriction only applies to R5-tropic viruses. In contrast, MVC is completely ineffective against HIV-1 if the virus instead enters the cell via the CXCR4 pathway. Both X4 and R5/X4 dual tropic viruses share this property, whether one of them is the dominant strain using CCR5 and the other uses CXCR4. Therefore, it is necessary to determine the viral tropism prior to contemplating the utilization of MVC [76,77,78,79,80,81].

Fostemsavir (FTR) is a recently approved medication for HIV-positive individuals. FTR is a methyl phosphate prodrug that, to produce its active component, temsavir (TMR) must first be hydrolyzed. By attaching to the gp120 protein on the viral envelope and interfering with the process by which the virus attaches to the CD4 receptor on the host, TMR acts as an HIV-1 attachment inhibitor and blocks viral entrance. Similar to how C-C chemokine receptor type 5 (CCR5) antagonists and fusion inhibitors work, TMR intervenes before the coreceptor binding and membrane fusion stages. TMR’s activity against different HIV-1 populations is not dependent on the viral tropism of those populations because TMR attaches directly to gp120 of the virus, close to the CD4 receptor binding site, preventing the initial phase of viral entry, and not a host cell receptor. As a result of this process, gp120 is locked in a closed state that prevents the conformational shift required for initial engagement with the surface receptors on CD4+ cells, hence blocking attachment and entrance into host T cells and other immune cells [82,83,84,85].

Enfuvirtide (ENF) is the first anti-HIV medication based on a fusion inhibitory peptide that was authorized for use in clinical practice [86]. Enfuvirtide is able to prevent HIV from entering cells by interfering with the process of membrane fusion. HIV-mediated fusion of the viral membranes with those of the host cell membranes entails a complicated chain of events, and a sequence of conformational shifts appears in the glycoproteins gp41 and gp120 [87]. ENF is a peptide that is composed of 36 amino acids and corresponds to the subsequent heptad repeat (HR2) sequence of gp41. Both the HR-1 and HR-2 heptad repeat regions of gp41 are responsible for the self-assembly of the six-helix bundle, commonly referred to as the hairpin structure. The establishment of such an arrangement places the membranes of viruses and cells in close proximity to one another. It is generally believed that the energy that is produced as a result of this change in conformation is what makes the process of fusion possible. ENF acts similarly to HR-2 and is able to attach to the HR-1 region of gp41, stopping the six-helix bundle from forming, which in turn blocks the fusion process and prevents HIV-RNA from entering the cell [87,88,89,90].

To date, ibalizumab is the only approved monoclonal antibody for treating HIV-1 infection. By disrupting the post-attachment stages essential for viral entry, ibalizumab blocks viral transmission-mediated cell–cell fusion and prevents HIV-1 from entering CD4 cells. Ibalizumab blocks gp120-CD4 complex conformational alterations that allow for coreceptor attachment and fusion. Specifically, ibalizumab recognizes a region in the CD4 receptor’s subsequent domain on an outer surface that is in close proximity to the receptor’s binding sites for gp120 and major histocompatibility complex class II (MHC-II). Ibalizumab does not inhibit the MHC-II-mediated immune response. The syncytium that is formed by infected and uninfected CD4 cells due to HIV-1 infection is likewise stopped by ibalizumab [91,92,93,94,95,96].

Antiretroviral therapy with a prolonged half-life is the most recent advancement in HIV treatment drugs [97,98]. Every one to two months, a healthcare provider should administer cabotegravir-rilpivirine (CAB/RPV) via a pair of injections, one containing each of its active constituents [99]. Utilizing a long-acting injectable antiretroviral treatment (LAA) could serve as a viable alternative in the realm of HIV prevention and management [98]. There are several expectations, such as reduced dose frequency, decreased drug–drug interactions, and fewer side effects. Furthermore, prospective patients would anticipate enhanced safeguarding of health confidentiality, mitigating pill tiredness, and potentially enhancing adherence concerns [100]. The medications, which include cabotegravir, an effective INSTI, and rilpivirine, an effective NNRTI, have been the subject of intense research for LAA therapy [101,102]. This is primarily due to their extended half-lives and significant intrinsic antiretroviral efficacy. Cabotegravir and rilpivirine exhibit limited solubility in water, enabling their incorporation into wet-milled suspensions, which yields drug crystals of nanoscale purity that are stabilized by surfactants, resulting in a formulation that is appropriate for intramuscular depot administration [103]. Nevertheless, CAB/RPV has certain drawbacks, such as the need for substantial injection quantities, the potential for drug-resistant virus strains to emerge during sub-therapeutic exposure following treatment cessation, and the restricted ability of native CAB and RPV to reach cellular and tissue reservoirs of infection [104]. 

## 4. HIV Drug Resistance Data

**Figure 4:**
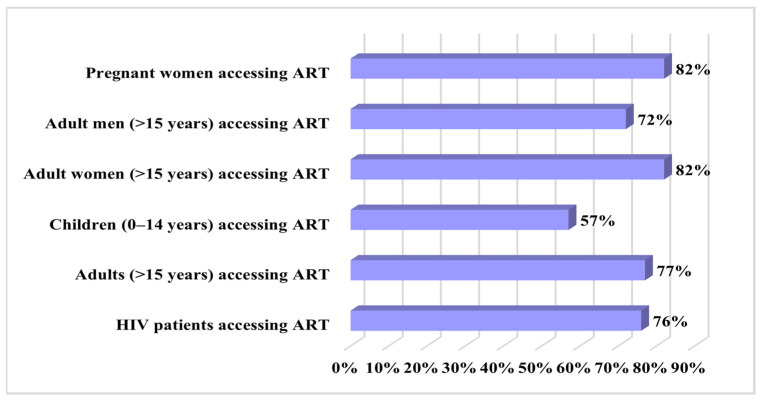
Global demographic distribution of patients with access to antiretroviral therapy in 2023 (data from [105]).

According to data published by UNAIDS, in 2023, there were approximately 39 million HIV-positive individuals, with just 29.8 million having access to antiretroviral treatment [105]. The demographic distribution of patients with access to antiretroviral therapy at the end of 2023 is depicted in Figure 4.

**Figure 5:**
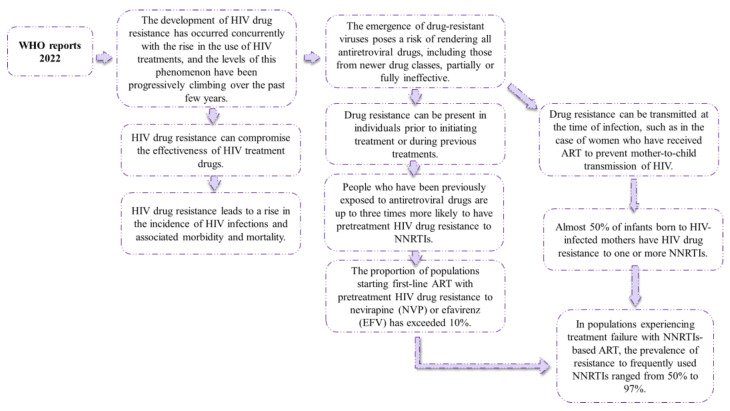
WHO reports on antiretroviral medication resistance (Adapted from [106]).

**Figure 6:**
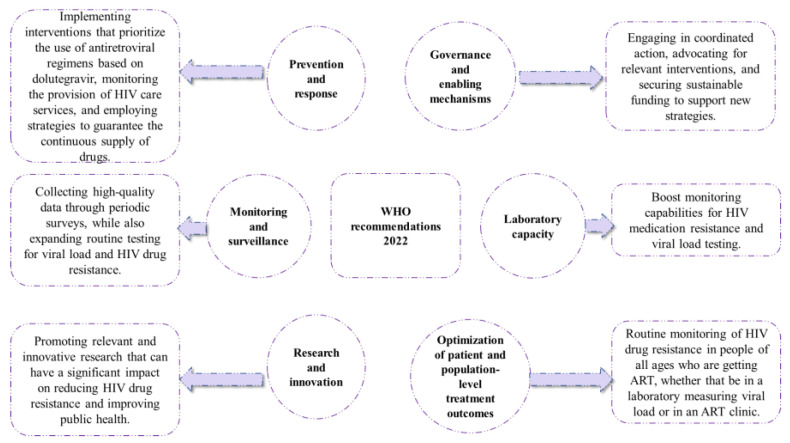
WHO recommendations for limiting antiretroviral resistance (Adapted from [106]).

ART has revolutionized the management of HIV/AIDS, leading to significant improvements in the lifespan and well-being of infected individuals. However, the effectiveness of these drugs can be compromised due to the emergence of ART resistance, which is driven by the high mutation rate of HIV. When individuals receive ART, it creates selective pressure on the virus, leading to the emergence of drug-resistant strains that can cause treatment failure, increased morbidity, and mortality. Therefore, monitoring ART resistance is crucial to identifying drug-resistant strains and guiding treatment strategies. The World Health Organization recommends routine surveillance of acquired HIV drug resistance to respond proactively to the rapidly evolving HIV epidemic and provide optimal care for those living with HIV/AIDS. Figure 5 depicts the WHO’s 2022 reports on antiretroviral resistance, and Figure 6 displays the WHO’s general recommendations to halt the accelerated evolution of resistance to HIV treatment drugs [106]. Figure 5 depicts the most recent WHO reports on antiretroviral medication resistance, whilst Figure 6 depicts WHO recommendations for limiting the spread of antiretroviral resistance.

## 5. HIV Drug Resistance Mechanisms

In the 1980s, when first-generation antiretrovirals were introduced, drug resistance was inevitable for all patients, and the time frame for effective therapy was brief. Some patients have been receiving care for an extended period without any resistance issues, whereas for other patients, resistance to HIV drugs possessed a hazard to their health. Even though ART has significantly decreased the morbidity and mortality associated with HIV infection and even though the use of a combination of ART can significantly improve HIV patients’ chances for long-term survival, the efficacy of antiretroviral drug therapy can be markedly reduced by the development of drug resistance. Resistance to antiretroviral drugs is a major contributing factor to the failure of treatment among HIV patients. Viruses that are resistant to various types of ART are common. This drug cross-resistance, combined with the frequently unpredictable emergence of drug resistance, poses significant challenges for HIV treatment. The successful treatment of HIV necessitates an in-depth comprehension of the mechanisms by which resistance may develop, as well as strategies for overcoming resistance once it occurs [107,108].

**Figure 7:**
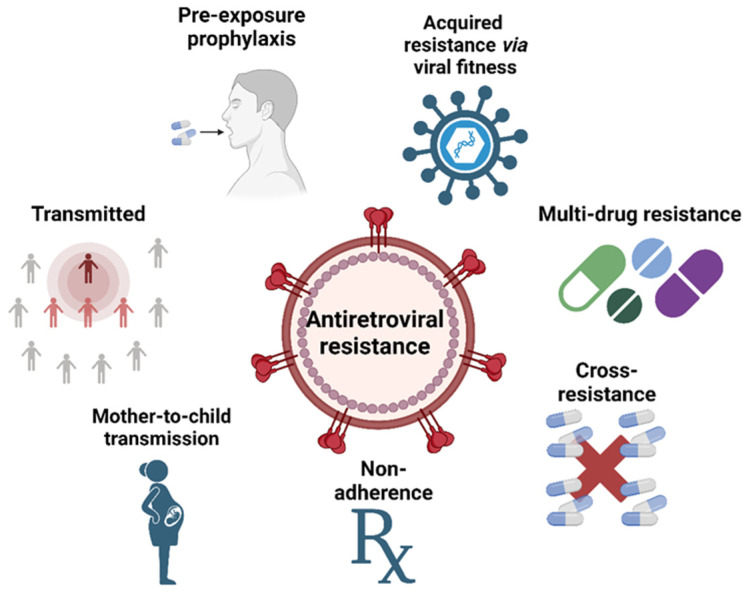
Causes leading to antiretroviral resistance (created with Biorender.com).

The high rate of replication and low fidelity of the reverse transcriptase enzyme in HIV contribute to the rapid and widespread emergence of drug resistance. HIV can produce billions of new viral particles each day, resulting in numerous variants with different sensitivities to antiretroviral drugs. Additional factors that may contribute to drug resistance include poor patient compliance, subtherapeutic drug levels, and inappropriate drug choice. Resistance may also develop as a result of inadequate exposure to antiretroviral agents, even during successful therapy [108]. Figure 7 summarizes the causes and factors implicated in the development of antiretroviral resistance.

### 5.1. Transmitted HIV Drug Resistance

Individuals oblivious to their HIV-positive status, those with a diagnosis with high concentrations of CD4+ cells who are ineligible for therapy, and ART-experienced patients with unsuppressed blood viraemia due to therapy failure are prone to spreading HIV [109,110,111,112,113]. According to WHO, the prevalence of medication resistance among people who did not respond to NNRTI-based first-line antiretroviral therapy (ART) varied between 50% and 97% [106].

Thus, roughly ten percent of new HIV-1 infections originate from strains resistant to traditional therapies [114,115], demonstrating that treated individuals are contributing to the spread of newly acquired infections. Moreover, individuals who initially become infected with drug-resistant strains are also able to pass on drug-resistant HIV [109,110,111,112,113]. This means that it is possible for drug-resistant strains of HIV to be transmitted from one patient to another, which can result in a newly infected patient carrying a drug-resistant virus, even if they have not yet received antiretroviral therapy. In other words, the virus may have developed resistance to certain drugs in a previously infected individual and can be passed on to a newly infected individual, potentially making their treatment more challenging [106,110].

In eastern and southern Africa, where the frequency of transmitted drug resistance has been estimated to be approximately 7%, the spread of ART in nations with middle and low incomes has led to a substantial rise in transmitted drug resistance. In wealthy countries, genotyping the virus before commencing therapy is a routine procedure, allowing clinicians to select an appropriately active combination of medications to attain high treatment success rates for individuals with transmitted drug resistance. Patients who have acquired medication resistance may begin treatment with ART regimens that are not robust enough, which can lead to the development of resistance in many drug classes in low-income countries where viral genotyping is typically unavailable. Furthermore, patients in countries with lower incomes have fewer second-line therapy alternatives, increasing the burden of transferred medication resistance [107,116,117,118].

### 5.2. Acquired HIV Drug Resistance via Viral Mutations

**Figure 8:**
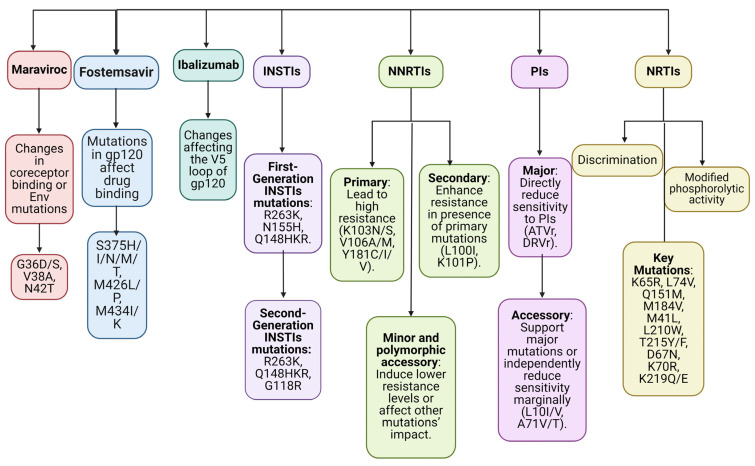
Drug classes and resistance mutations (created with Biorender.com).

Patients on ART show the most consistent tendency to develop acquired drug resistance in the form of a continuously increasing percentage of patients with medication resistance as therapy progresses [107]. In a study by Mafalda et al., 26,973 people living with HIV-1 were included; 11,581 (or 42.9%) had never had antiretroviral therapy before, while 15,392 (or 57.1%) had. There was a declining trend in the total prevalence of both transmitted and acquired drug resistance (12.8% and 68.5%, respectively). The rates of transmitted drug resistance were 12.3 and 12.6% among late vs. non-late presenters, whereas the rates of acquired drug resistance were 69.9 and 68.2% among the two groups. K103N/S, T215rev, T215FY, M184I/V, M41I/L, M46I/L, and L90M were the most often transmitted drug resistance variants in both late and non-late presenters [119]. In a meta-analysis of trials in patients from high-income nations conducted by Stadeli et al., HIV drug resistance was detected in 7% of patients after 6–11 months of ART, 11% after 12–23 months, and 21% after 36 months or longer [120]. Another investigation conducted in the UK by Phillips et al. found that after four, six, and eight years of NNRTI-based treatment, the percentage of patients having at least one drug-resistance mutation rose from 11% to 14%, 16%, and 18%, respectively. According to the results of this study, even if a patient’s viral population has not evolved resistance after six years of therapy, there is still a chance that it will do so during the seventh year of treatment at a rate of around 2% [116]. Hauser et al. conducted a comprehensive evaluation that included 2690 people living with HIV from 19 different research groups. After 2 years, a large percentage of patients who had failed first-line ART with emtricitabine or lamivudine had developed the M184V/I mutation, 75.7% if treated with tenofovir and 72.1% with zidovudine. K65R mutation frequency was 52.0% at 2 years with tenofovir disoproxil fumarate. The most common NNRTI resistance mutation on efavirenz was K103, followed by V106 [121]. Figure 8 illustrates an overview of the most common viral mutations.

NRTI resistance manifests itself through two mechanisms, including discrimination and modified phosphorolytic activity of reverse transcriptase. The “discrimination” mechanism occurs when the reverse transcriptase enzyme avoids attaching to the NRTI but still recognizes the natural deoxynucleoside triphosphate (dNTP) substrate. K65R, L74V, Q151M, and M184V are all examples of point mutations in HIV that decrease the affinity of reverse transcriptase for a particular NRTI without affecting its affinity for the matching dNTP substrate. The end result is a reduced drug uptake into the DNA strand. Increased drug clearance from the point of attachment at the DNA chain terminus is the second mechanism of NRTI resistance. These modifications to the reverse transcriptase open up the active site next to the bound nucleoside analog, making it possible for ATP or pyrophosphate to attach there. Because of the high energy of ATP or pyrophosphate, they are able to break the link holding the medication to DNA, releasing it and ending its effect. Thymidine analog mutations are M41L, L210W, T215Y, D67N, K70R, T215F, and K219Q/E. [108,122,123,124,125,126,127].

Resistance to NNRTIs develops mostly as a result of mutations in hydrophobic RT residues found in the binding pocket for NNRTIs. Mutations in the same location of RT that all of the NNRTIs bind to will have an effect on the binding of all of the drugs in this class. The substantial prevalence of HIV cross-resistance among members of this class of agents may be partially explained by this phenomenon [128]. Although NNRTIs tend to have fewer adverse effects than nucleoside analogues, their fast resistance to treatment is a major downside. Therefore, NNRTIs are never used as an independent treatment for HIV infection. Only one or two mutations are needed for high-level resistance to NNRTIs; hence, the genetic barrier to resistance is modest. In addition to the fact that many NNRTI mutations that confer resistance decrease susceptibility to multiple NNRTI, the relatively small genetic barrier to resistance means that a single NNRTI is able to opt for several NNRTI resistance mutations across various viruses, despite the fact that just one mutation can be identified by standard population-based sequencing, leading to elevated levels of clinical cross-resistance between different NNRTI [129,130,131].

NNRTI resistance mutations are divided into four types, including major, secondary, minor non-polymorphic, and polymorphic accessory mutations. Primary NNRTI resistance mutations emerge first during NNRTI treatment and are responsible for acquiring high degrees of resistance to one or more NNRTIs. Variable resistance to efavirenz and high resistance to nevirapine are caused by the following mutations: K103N/S, V106A/M, Y181C/I/V, Y188L/C/H, and G190A/S/E [132,133,134]. The choice of an NNRTI, especially etravirine, is greatly influenced by the presence of secondary NNRTI resistance mutations, which typically arise in tandem with the main NNRTI resistance mutations, including L100I, K101P, P225H, F227L, M230L, and K238T [132,133,134,135]. Polymorphic accessory mutations regulate the impact of other NNRTI resistance mutations, whereas minor non-polymorphic changes induce low-level decreases in susceptibility. Common variants that lower sensitivity to NNRTIs, like nevirapine and efavirenz, include A98G, K101E, V108I, and V179D/E [136]. Suppressing sensitivity to nevirapine and efavirenz by about twofold, highly polymorphic RT mutations like K101Q, I135T/M, V179I, and L283I may function synergistically with primary NNRTI resistance mutations. In addition to being largely selected by NRTI, mutations like L74V, H221Y, K223E/Q, L228H/R, and N348I also lead to modest decreases in sensitivity to NNRTIs [137,138].

Substrate-binding cleft substitutions in viral proteases are often the initial step toward resistance to protease inhibitors. These drug-resistant variants of the viral protease have an expanded catalytic site. This results in less binding to the inhibitor (and hence less drug sensitivity) and, concomitantly, less affinity to the natural substrate (and thus less viral multiplication). Primary or “major” resistance mutations are those that are selected initially and individually lower a cell’s sensitivity to a protease inhibitor. Secondary or “minor” mutations appear subsequently and have little to no influence on the resistance phenotype on their own, but they boost viral replication when combined with viruses harboring large alterations. HIV-1 clades that are not of the subtype B variety share a small number of mutations that occur as polymorphisms [139,140,141]. The protease inhibitor class is responsible for more mutation selection than any other antiretroviral drug class. When many mutations in PI resistance are present in the same viral isolate or when mutations occur in an unexpected manner, it may be difficult to accurately assess the impact of each mutation on a given PI. Most protease inhibitor resistance mutations are “accessory”, meaning that they only work to counteract the replication impairment caused by other protease inhibitor resistance mutations or to decrease protease inhibitor susceptibility when used in conjunction with additional resistance variants [132]. Major protease inhibitor resistance mutations are ATVr, DRVr, FPVr, IDVr, LPVr, NFV, SQVr, and TPVr. Accessory protease inhibitor resistance mutations are L10I/V, A71V/T, I13V, D60E, I62V, V77I, and I93L [127].

It has been determined that the N155H, Q148K/R/H, and Y143R/C substitutions are the major mutations responsible for resistance to first-generation INSTIs [142,143,144]. Second-generation INSTIs have more substantial resistance than their first-generation counterparts [145,146,147]. To block the catalytic divalent metal ion-dependent phosphodiesterase (DDE) triad [48,148,149,150], INSTIs possess a halogenated phenyl group that attacks the catalytic pocket and shifts the 3′ viral end [151,152], as well as three coplanar oxygen atoms that bind to the divalent metal ions within the catalytic pocket. INSTIs are strand transfer-specific and only weakly impede 3′ processing [75,153], despite the fact that the coordination of the metal ions by the triad is also required for 3′ processing. This problem arises because of an allosteric barrier that exists between the halogenated phenyl group and the 3′ dinucleotide that is cleaved during 3′ processing [151,152]. This hindrance limits the effective binding of INSTIs before 3′ processing takes place. Most mutations that confer resistance to INSTIs are located in the catalytic core domain, close to the catalytic pocket where the DDE triad is situated [154]. Table 2 summarizes resistance mutations for INSTIs.

Entry inhibitors directly target the extremely changeable HIV Env protein. The wide range of susceptibility of HIV-1 strains to entry inhibitors at baseline is probably attributable to differences in Env [161]. The expression levels of coreceptors and the degree of attraction with which Env binds to its coreceptors are two factors that affect susceptibility to entry inhibitors [162,163]. The variability of the HIV genes is most pronounced in the *env* gene, which can result in differences in susceptibility to HIV medication [164].

The time that the ENF-binding site on HR1 is exposed decreases in correlation with the amount of coreceptor affinity or expression [162,163]. Resistance to ENF can emerge in the face of selective drug pressure due to HIV’s rapid reproduction rate and the poor accuracy of the reverse transcriptase enzyme [165,166,167,168]. Modifications in the amino acid triad (GIV) at positions 36–38 in the HR1 region of gp41 have been linked to variations in sensitivity and the emergence of resistance. Supporting the idea that HR2-HR1 binding is necessary for fusion and that HR2 homologues like enfuvirtide function by inhibiting this connection, mutations in this region have occurred under selection pressure [166,169,170]. Amino acid positions 36–45 have been linked to a wide range of mutations, each of which confers a particular degree of resistance or sensitivity to ENF in a specific molecular setting. The majority of mutations in this area are single amino acid changes, which result in various levels of susceptibility loss [167,168,169]. It is also possible for mutations to occur in a serial manner when the reverse of the first mutation occurs at the same time as the onset of the second. Mutations in the amino acid region 36–45 are NL4-3G, G36D, G36S, V38A, Q40H, N42T, N42E, N42S, N43D, N43S, N43K, L44M, L45M, G36S + L44M, N42T + N43K, N42T + N43S, V38A + N42D, V38A + N42T, and V38E + N42S [171]. According to a study by Leung et al. [172], it is crucial to test for mutations before beginning ENF therapy due to the high occurrence of ENF resistance-associated mutations in both ART-experienced and ART-naive individuals. There was still evidence of the G36D mutation six months after the removal of ENF. 

MVC, a drug also targeting the *env* gene, has proven to be a challenging case in terms of resistance development [164]. MVC resistance can occur through either tropism change or mutations that allow HIV-1 to continue using CCR5 coreceptors, despite the presence of the drug [173]. Most viruses that have developed resistance to MVC have maximal percentage inhibition values between 80% and 95%, indicating that this resistance is noncompetitive [174,175]. Through mutations that boost gp120’s affinity for MVC-bound CCR5, resistance to MVC can arise, allowing gp120 to connect to CCR5, ignoring conformational alterations caused by MVC binding [176]. In the presence of MVC, viruses with resistance may interact with CCR5 by binding more strongly to the CCR5 N-terminal domain [175]. Modifications in the V3 loop might enhance the virus’s affinity for the location where it binds, allowing it to connect to the extracellular loop, even in the presence of MVC, which is another explanation for resistance to MVC [176]. Ray N. [164] suggested several potential mechanisms for MVC resistance, including more efficient scavenging of unbound coreceptors, competing with drug-bound coreceptors, using the drug-bound coreceptor, switching to using CXCR4 or preexisting CXCR4 virus, and shifting to alternative coreceptors (such as CCR2, CCR8, etc.).

Patients with HIV-1 who have developed resistance to many drugs now have a new option in the form of FTR. No modifications to the dosage for renal function or liver function are necessary, and it is usually well tolerated [177]. Several investigations into the FTR resistance problem have been carried out. While there was no direct correlation between amino acid substitutions in gp-120 (S375H/I/N/M/T, M426L/P, M434I/K, and M475I) and the emergence of resistance to FTR in in vivo studies, these variations in EC50 values suggested that they could affect HIV’s susceptibility to this drug [178,179,180]. The incidence of aminoacidic substitution was modest in a retrospective investigation of gp-120 mutations in 409 treatment-naive HIV-positive patients. The most common mutation was S375T, which presumably plays a secondary role in the impairment of FTR susceptibility, leading to the conclusion that viruses that have this single mutation have a high treatment susceptibility [181]. Other studies [85,179,182,183] have shown that some substitutions, particularly the ones positioned within or near the site of binding of FTR and/or of CD4, can lead to diminished hydrophobic bonds among the drug and gp120 or can decrease the dimensions of the FTR binding site, thus restricting FTR’s ability to access their binding sites and resulting in resistance or diminished susceptibility in vitro. Seven mutations have been found to lower fostemsavir susceptibility in vitro, including L116P/Q, A204D, S375M/H/T, M426L, M434I, S475I, and V506M) [85,184,185]. All these mutations are within the gp120 area. Experiments performed in vivo have helped pinpoint a few of these variants, including S375M/T, M426L, M434I, and S475I [179,185]. In patients who have never used fostemsavir, only a small number of studies have reported primary resistance to the drug in the HIV-1 B subtype, but the total number of sequences examined was modest [181,186,187]. According to reports, FTR is able to effectively target the majority of HIV-1 subtypes; resistance mutations are rare and do not result in cross-resistance with other classes of antiretroviral drugs. This suggests that it could be used alongside currently available drugs [181,186,187,188,189,190,191,192].

Ibalizumab’s therapeutic activity can be blocked by resistance mechanisms that weaken his bond with the CD4 cell receptor [93,193,194]. The V5 loop is a visible part of gp120’s exterior. It appears that the key mechanisms underlying resistance to ibalizumab are the reduced expression or deletion of V5 potential N-linked glycosylation site (PNGS) and the particular locations of PNGS [93,193]. V5 PNGS variability was shown to correlate with susceptibility, with the lack of PNGS conferring the greatest resistance. Site 1 deletion was shown to be the most common in resistant versions, and removing other parts of PNGS also altered the susceptibility [93,193,195].

The presence of drug-resistance mutations in patients receiving long-acting cabotegravir and rilpivirine poses a significant risk for virological failure [196,197]. This treatment protocol is advised for patients who have achieved virological suppression and do not have failed therapies or potential resistance, except for isolated K103N. Steegen et al. conducted a retrospective analysis that showed that rilpivirine drug resistance mutations were frequently employed in a context in which NNRTI-based therapy was commonly used. A total of 12.3% of patients starting ART had a minimum of one rilpivirine drug resistance mutation [196]. In a multiple-case analysis conducted by van Welzen et al., five patients considered eligible for CAB/RPV developed virologic failure, despite having a low projected risk at baseline. The genotypic resistance testing demonstrated a significant prevalence of mutations linked with NNRTI in all instances, while INSTI mutations were observed in only four cases. During the course of treatment, it was observed that all cases exhibited diminished levels of either CAB, RPV, or both drugs, which is likely a contributing factor to the manifestation of virologic failure [197]. Moreover, there has been a noticeable rise in cabotegravir resistance in both the 2-month and 1-month treatment plans, indicating the need for a thorough evaluation of the advantages and disadvantages when choosing between the 1-month or 2-month treatment plan for specific patients [99]. The implementation of targeted genotyping may be necessary for patients who are commencing CAB/RPV, hence introducing a notable level of complexity to the existing public health strategy [196]. The use of CAB/RPV may not be suitable for all HIV-positive patients [99,198]. In cases in which individuals demonstrate resistance, conventional ART regimens may be the sole viable approach for attaining viral suppression at present. Eligibility requirements for patients to undergo CAB/RPV consist of a recorded viral load that cannot be detected, a well-documented lack of resistance to both CAB and RPV, and no previous instances of failure in antiretroviral treatment [99].

### 5.3. Cross-Resistance

Cross-drug resistance among antiretroviral agents of the same class is a prevalent issue that impacts all main classes. An exponential increase in the incidence of HIV drug resistance among ART-naive individuals poses a significant barrier to eradicating the HIV-1 outbreak by 2030. Non-nucleoside reverse transcriptase inhibitor (NNRTI) resistance affects 10% of adults initiating HIV treatment. Individuals with a history of antiretroviral medication use are three times more likely to exhibit resistance. It is estimated that the prevalence of drug-resistant viruses in classes three and four is between 5 and 10% in Europe, whereas in North America, it is below 3% [114].

The utilization of NNRTIs in HAART is crucial, owing to their limited adverse effects and toxicity profiles. Nevertheless, the efficacy of these interventions may be compromised by resistance and cross-resistance. NNRTI resistance is influenced by factors such as variations in the sequence of residues that align the NBP, as well as the effects of mutations that confer resistance on the sensitivity to drugs and the fitness of the virus [199].

Research has demonstrated that the buildup of thymidine analogue-resistant mutations is responsible for nearly all resistance to NRTIs. The M184V mutation exhibited the highest prevalence among resistance variants in NRTIs, with the K65R mutation following closely after. There is evidence suggesting that the M184V mutation confers substantial resistance to 3TC while concurrently enhancing susceptibility to TDF and ZDV. According to Lin et al., the K65R mutation has been found to result in a significant level of resistance to TDF and ABC [200].

Tran-To Su et al. identified a PI resistance-related pathway through which HIV protease might progress in PI cross-resistance, determined by the structural modifications and viral fitness impact of the mutations that have been clinically reported. The structural justification for the swift emergence of cross-resistance was found in five out of the seven PIs that are utilized clinical practice [201]. Rhee et al. emphasized that nonpolymorphic mutations have a more significant effect on decreased PI susceptibility than polymorphic mutations. I84AV, V32I, G48V, I54ALMSTV, V82F, and L90M were found to be related to decreased susceptibility to eight PIs. Among the mutations that had the most significant impact on PI susceptibility, I47A, G48M, I50V, L76V, V82ST, and N88S were all linked to reduced susceptibility to a range of four to five PIs. Lastly, D30N, I50L, and V82AL were correlated with lowered susceptibility to less than four PIs [202]. 

However, the very first approved monoclonal antibody employed for the treatment of HIV-1 infection, ibalizumab, presents great promise for overcoming the cross-resistance issue. No cross-resistance between ibalizumab and any antiretroviral drugs (such as NRTIs, NNRTIs, PIs, INSTIs, or entry inhibitors) was found using phenotypic and genotypic testing [96,191,203]. The sensitivity of HIV-1 polymorphisms linked with enfuvirtide resistance (such as G36D, V38A, and N43D) to ibalizumab was shown in vitro. Likewise, HIV strains that were resistant to ibalizumab continued to exhibit susceptibility to enfuvirtide [204].

In summary, the occurrence of cross-resistance in antiretroviral drugs serves as an illustration of the complex dynamics of HIV viral evolution and emphasizes the necessity for an all-encompassing strategy that incorporates molecular investigation and the discovery of novel chemical structures that can be employed to address this phenomenon.

### 5.4. Multidrug Resistance (MDR HIV)

MDR HIV is a term used to describe HIV strains that have decreased susceptibility to medications in all three categories of antiretroviral treatments [205]. Individuals afflicted with multidrug-resistant infection with HIV-1 are prone to treatment ineffectiveness, experience deteriorated clinical results, and face an elevated mortality risk compared to other patients infected with HIV-1 [204].

In present time, there are two drugs used for ART experienced patients, including fostemsavir and ibalizumab. Fostemsavir, an oral prodrug classified as an HIV-1 attachment inhibitor, is recommended for use in conjunction with other antiretroviral medications to address HIV-1 infection in adults with a history of extensive treatment and MDR HIV infection. This is particularly relevant for individuals whose current antiretroviral regimen proves ineffective due to resistance, intolerance, or safety concerns [83]. Ibalizumab, when administered intravenously twice monthly, is approved for the treatment of extensively treatment-experienced persons with multidrug-resistant HIV-1 infection who have few or no other treatment options [114].

### 5.5. Nonadherence in Drug Resistance

According to one definition, medication adherence refers to “the degree to which patients take medications in accordance with the directions given to them by their healthcare providers” [206].

Adherence to antiretroviral medication has been recognized as a major factor in HIV treatment success. To reduce the risk of developing drug resistance, slow the development of illness, and shorten the lifespan of those living with HIV, it is essential that patients adhere to ART as prescribed. Genetic predisposition, behavior, lifestyle, and societal or structural concerns are some of the variables that influence the successful treatment of HIV infection [207,208]. Another factor influencing adherence is the individual susceptibility to pharmacological side effects. Predictors of behavior consist of elements like conforming to a schedule. The stigma associated with HIV illness in the local community is one of the social difficulties. Establishing and making accessible effective drugs, establishing criteria for the appropriate administration of these treatments, and ensuring consistent patient accessibility to medical services and drugs are all examples of structural difficulties in the healthcare system [209,210,211,212].

### 5.6. Mother-to-Child Drug Resistance

HIV is transmitted from mother to child mostly during the third trimester of pregnancy, or the brief time period between placental separation, labor, and delivery. The rates of transmission without preventative measures range from 15% to 40%. In addition to suppressing viral replication, antiretroviral medications also have an autonomous effect that lowers the risk of HIV transmission through the plasma and the vaginal tract. Antiretroviral drug transfer through the placenta is one method of HIV transmission prevention. Prolonged maternal dosage throughout pregnancy and labor results in detectable antiretroviral medication levels in the newborn plasma shortly after delivery. Since 2002, the existence of mutations in HIV strains suggestive of ART resistance has been advised to be screened for prior to treatment beginning in HIV-infected pregnant women [213,214].

In areas with limited medical resources, it is common practice to administer treatment to pregnant women to reduce the risk of HIV being passed from the mother to the child. Nevirapine (an NNRTI) is the most straightforward method. While a single dose of nevirapine can decrease the likelihood that a child could contract HIV during pregnancy, it also carries a substantial risk of medication resistance in both the mother and the child [107]. Arrivé et al. [215] conducted a meta-analysis and showed that several weeks following treatment with single-dose nevirapine, on average, 36% of the mothers and 53% of the children had detectable levels of nevirapine resistance. Pennings [216] proposed that the significant risk of resistance to single-dose nevirapine was attributable to the presence of a large number of NNRTI mutations prior to treatment. 

During the years 2010–2012, another study found that 51% of HIV infants had developed drug resistance to HIV-1 medications, especially to NNRTIs. The greatest frequency of resistance (74%) was seen in newborns whose mothers’ received ART, while 26% of HIV-positive infants with no or unverified ART treatment harbored NNRTI resistance. These findings show that NNRTI resistance is rising among infants with new HIV diagnoses in a high-HIV prevalence population in which coverage of ART among pregnant women has grown over time, both in terms of timing of initiation and CD4 cell count at the time of conception [217]. Maternal HIV drug resistance and viral load were found to be independent risk factors for vertical transmission during breastfeeding, according to an analysis by Boyce et al. [218] of 85 cases and 255 matched controls, suggesting that nevirapine alone may not be adequate infant prophylaxis against drug-resistant variants in maternal breast milk.

### 5.7. Preexposure Prophylaxis

Preexposure prophylaxis (PrEP) is an efficacious strategy for mitigating the transmission of HIV. It consists of daily administration of an antiretroviral tablet and adherence to additional preventive behavioral measures [219]. This preventive mechanism is employed for those who have not received a formal diagnosis of HIV but may face a significant risk of contracting the virus due to their lifestyle or involvement in a serodiscordant relationship [220].

The findings derived from clinical trials provide evidence of the effectiveness of PrEP, whether employed as a standalone intervention or in conjunction with other techniques for prevention. These trials have consistently demonstrated that PrEP can significantly decrease the occurrence of HIV by as much as 86%, and in some cases, even further with rigorous adherence [220,221,222].

Multiple clinical trials have provided clear evidence of the efficacy of a fixed-dose combination of TDF/3TC, as recommended by the WHO, in mitigating the risk of HIV transmission. The WHO has recently proposed the use of this strategy as a means to address the issue of newly diagnosed HIV infections amongst those who are at significant risk of contracting HIV, such as commercial sex workers and discordant individuals. When taken on a daily basis by a seronegative individual, PrEP offers a decrease of over 90% in the acquisition of HIV [223].

Nevertheless, the possibility of PrEP contributing to the development of antiretroviral resistance continues to be a significant concern in the field of public health. Empirical data indicate that the occurrence of HIV medication resistance selection through PrEP usage is rare and is most probable when PrEP is employed during an undetected early HIV infection [224].

## 6. Challenges and Opportunities in Overcoming HIV Drug Resistance

Oral PrEP and combination antiretroviral medication are essential components in combating the HIV epidemic. Nevertheless, despite their efficacy, improved tolerability, and enhanced convenience, these medications possess certain limitations that can potentially impact both quality of life and adherence. These limitations include the fatigue associated with daily long-term treatment, the challenges faced by certain patients in swallowing tablets, and the possibility of drug interactions with other medications being taken simultaneously [100,225].

As of 2021, CAB/RPV is the first long-acting injectable antiretroviral therapy authorized for use in HIV patients who are treatment-naive or experienced [99,226]. Moreover, there has been a notable surge in research on the creation of long-acting pharmacological formulations for people diagnosed with HIV [104,227]. In addition to CAB/RPV, research focuses on alternative long-acting injectables, including long-active capsid inhibitors and long-acting slow-effective release antiretroviral therapy (LASER ART). LASER ART consists of uniform particles of solid prodrug nanocrystals that are stabilized by aqueous surfactant formulations [104,228].

Furthermore, there is ongoing research on additional pharmaceutical formulations aimed at improving the efficacy of HIV treatment protocols through the enhancement of the bioavailability of the active ingredient, promotion of adherence, and mitigation of adverse effects [229]. These consist of extended-release oral medications [230], formulations containing oral nanoparticles [231], transdermal patches, and microneedles with extended-release properties [232], as well as vaginal and rectal microbicides [233]. 

In the clinical arena, there is a strong pursuit of passive immunization techniques that employ robust broadly neutralizing antibodies to prevent the transmission of HIV-1, as there is currently no effective vaccine available for the prevention of HIV-1 infection [234]. Neutralizing antibodies targeting Env have the ability to impede viral entry and hinder infection by disrupting the interaction with CD4, CCR5, or CXCR4. This is achieved through the stabilization of prefusion Env, which prevents membrane fusion, or by promoting the degradation of Env [235].

The primary objectives of the new drug development strategies are to enhance the safety and resistance profile of established antiretroviral classes, identify drugs that operate through novel mechanisms (e.g., capsid inhibitors, nucleoside reverse transcriptase translocation inhibitors, attachment/post-attachment inhibitors), combine therapies that promote better adherence, and simplify treatment through infrequent dosing [114]. If these efforts prove to be effective and gain widespread acceptance among healthcare professionals and patients, they might offer more advanced and streamlined alternatives for the prevention and management of HIV infection [104].

## 7. Conclusions

Health systems worldwide are facing ongoing challenges due to the evolution of the human immunodeficiency virus and the concerning rise in resistance to antiretroviral medication. Comprehension and awareness of the mechanisms through which resistance to antiretroviral therapy arises motivate the collaborative attempt to combat HIV/AIDS and enhance treatment results and health equality.

## Figures and Tables

**Table 1:** HIV drugs’ adverse reactions (Adapted from [52,53,54,55,56,57,58]).

Classification	Mechanism	Nervous System	Cardiovascular	Digestive System	Liver	Renal	Skin	Metabolism	Other
NRTIs: emtricitabine, zidovudine, didanosine, stavudine, abacavir, lamivudin, tenofovir alafenamide, tenofovir disoproxil fumarate	reverse transcriptase inhibition by competing with natural nucleosides	headache, peripheral neuropathy	cardiomyopathy	nausea, diarrhea, pancreatitis	hepatic steatosis	Fanconi’s syndrome, renal insufficiency	rash	lactic acidosis, lipodystrophy, hyperlactatemia	hypersensitivity reaction, lipoatrophy, thrombocytopenia, anemia, myelosuppression
NNRTIs: nevirapine, delavirdine, efavirenz, etravirine, rilpivirine, doravirine	reverse transcriptase polymerization inhibition by binding to the NNRTI binding pocket	depression, sleep disturbance, headache		nausea, diarrhea	hepatitis		rash	dyslipidemia	neutropenia
INSTIs: bictegravir, dolutegravir, elvitegravir, raltegravir, cabotegravir	integrase inhibition	intracranial hemorrhage, sleep disturbance, headache	myocardial infarction	nausea, diarrhea	unconjugated hyperbilirubinemia	nephrolithiasis, renal insufficiency	rash	weight gain	myopathy, rhabdomyolysis
PIs: atazanavir, darunavir, fosamprenavir, indinavir, lopinavir, nelfinavir, ritonavir, saquinavir, tipranavir	protease inhibition	dizziness, headache	QT prolongation	nausea, diarrhea	hepatotoxicity	nephrolithiasis, renal insufficiency	rash	dyslipidemia, hyperglycemia, fat maldistribution	
Maraviroc	binds to CCR5, prevents the interaction of HIV-1 gp120 with CCR5-tropic HIV-1, and inhibits the virus from entering the cell	dizziness, headache	postural hypotension		hepatitis	renal insufficiency			
Fostemsavir	(prodrug) binds the viral envelope protein gp120 on HIV-1, prevents the conformational change required for attachment of HIV-1 to the host cell	headache, neuropathy	QT prolongation	nausea, vomiting, abdominal pain, diarrhea			rash		myalgia
Enfuvirtide	binds to HR1 in the gp41 subunit of the viral envelope glycoprotein, prevents the conformational changes required for the fusion of viral and cellular membranes	dizziness, headache, sleep disturbance		nausea, diarrhea			rash		injection site nodules, hypersensitivity
Ibalizumab	blocks gp120-CD4 complex conformational alterations that allow for co-receptor attachment and fusion	dizziness, headache		nausea, diarrhea					immune system disorders
Long-acting injectable antiretroviral drugs (cabotegravir-rilpivirine)	INSTI + NNRTI	pyrexia, headache, sleep disorders, dizziness, depression		nausea	abnormalities in aspartate aminotransferase, alanine aminotransferase, total bilirubin		rash		fatigue, injection site reactions, musculoskeletal pain

**Table 2:** INSTIs resistance mutations (Adapted from [155]).

	Drug	Mutation	
		Major	Accessory
First-generation INSTI	Raltegravir [156]	R263K, N155H, Q148HKR, Y143RHC, F121Y	L74M, E138AK, G140AS, E92Q, T97A
	Elvitegravir [157,158]	R263K, N155H, Q148HKR, S147G, F121Y, E92Q, T66I	T97A, E92G, T66AK
Second-generation INSTI	Dolutegravir [159]	R263K, Q148HKR, G118R	N155H, G140AS, E138AKT, T66K, E92Q, F121Y
	Bictegravir [160]	R263K, Q148H	G140S, E138K, T66K, E92Q, G118R
	Cabotegravir [160]	R263K, Q148HKR, G140R, G118R	N155H, S153FY, G140ACS, E138AKT, T66K

## References

[1] The Joint United Nations Programme on HIV/AIDS (UNAIDS) (2022). Aids in Danger: UNAIDS Global AIDS Update 2022.

[2] Faria N.R., Rambaut A., Suchard M.A., Baele G., Bedford T., Ward M.J., Tatem A.J., Sousa J.D., Arinaminpathy N., Pépin J. (2014). The Early Spread and Epidemic Ignition of HIV-1 in Human Populations. Science.

[3] Nastri B.M., Pagliano P., Zannella C., Folliero V., Masullo A., Rinaldi L., Galdiero M., Franci G. (2023). HIV and Drug-Resistant Subtypes. Microorganisms.

[4] Nyamweya S., Hegedus A., Jaye A., Rowland-Jones S., Flanagan K.L., Macallan D.C. (2013). Comparing HIV-1 and HIV-2 Infection: Lessons for Viral Immunopathogenesis. Rev. Med. Virol..

[5] Esbjörnsson J., Jansson M., Jespersen S., Månsson F., Hønge B.L., Lindman J., Medina C., da Silva Z.J., Norrgren H., Medstrand P. (2019). HIV-2 as a Model to Identify a Functional HIV Cure. AIDS Res. Ther..

[6] Rawson J.M.O., Landman S.R., Reilly C.S., Mansky L.M. (2015). HIV-1 and HIV-2 Exhibit Similar Mutation Frequencies and Spectra in the Absence of G-to-A Hypermutation. Retrovirology.

[7] McCutchan F.E. (2000). Understanding the Genetic Diversity of HIV-1. AIDS.

[8] Korber B., Gaschen B., Yusim K., Thakallapally R., Kesmir C., Detours V. (2001). Evolutionary and Immunological Implications of Contemporary HIV-1 Variation. Br. Med. Bull..

[9] Oguntibeju O. (2012). Quality of Life of People Living with HIV and AIDS and Antiretroviral Therapy. HIV/AIDS—Res. Palliat. Care.

[10] Gupta A., Verma A., Kashyap M., Gautam P. (2020). ART in Prevention of Mother-to-Child Transmission of HIV. J. Obstet. Gynecol. India.

[11] Mugwaneza P., Lyambabaje A., Umubyeyi A., Humuza J., Tsague L., Mwanyumba F., Mutabazi V., Nsanzimana S., Ribakare M., Irakoze A. (2018). Impact of Maternal ART on Mother-to-Child Transmission (MTCT) of HIV at Six Weeks Postpartum in Rwanda. BMC Public Health.

[12] Ciaranello A.L., Seage G.R., Freedberg K.A., Weinstein M.C., Lockman S., Walensky R.P. (2008). Antiretroviral Drugs for Preventing Mother-to-Child Transmission of HIV in Sub-Saharan Africa: Balancing Efficacy and Infant Toxicity. AIDS.

[13] Campbell E.M., Hope T.J. (2015). HIV-1 Capsid: The Multifaceted Key Player in HIV-1 Infection. Nat. Rev. Microbiol..

[14] Sakuragi J.I. (2011). Morphogenesis of the Infectious HIV-1 Virion. Front. Microbiol..

[15] Fanales-Belasio E., Raimondo M., Suligoi B., Buttò S. (2010). HIV Virology and Pathogenetic Mechanisms of Infection: A Brief Overview. Ann. Ist. Super. Sanita.

[16] Kalinichenko S., Komkov D., Mazurov D. (2022). HIV-1 and HTLV-1 Transmission Modes: Mechanisms and Importance for Virus Spread. Viruses.

[17] Seitz R. (2016). Human Immunodeficiency Virus (HIV). Transfus. Med. Hemotherapy.

[18] Li G., Piampongsant S., Faria N.R., Voet A., Pineda-Peña A.-C., Khouri R., Lemey P., Vandamme A.-M., Theys K. (2015). An Integrated Map of HIV Genome-Wide Variation from a Population Perspective. Retrovirology.

[19] van Heuvel Y., Schatz S., Rosengarten J.F., Stitz J. (2022). Infectious RNA: Human Immunodeficiency Virus (HIV) Biology, Therapeutic Intervention, and the Quest for a Vaccine. Toxins.

[20] Shah S., Alexaki A., Pirrone V., Dahiya S., Nonnemacher M.R., Wigdahl B. (2014). Functional Properties of the HIV-1 Long Terminal Repeat Containing Single-Nucleotide Polymorphisms in Sp Site III and CCAAT/Enhancer Binding Protein Site I. Virol. J..

[21] Levy J.A., Steele F.R. (1995). Hiv and the Pathogenesis of Aids. Nat. Med..

[22] Kuiken C., Foley B., Leitner T., Apetrei C., Hahn B., Mizrachi I., Mullins J., Rambaut A., Wolinsky S., Korber B. (2010). HIV Sequence Compendium 2010.

[23] Lu K., Heng X., Summers M.F. (2011). Structural Determinants and Mechanism of HIV-1 Genome Packaging. J. Mol. Biol..

[24] Clark E., Nava B., Caputi M. (2017). Tat Is a Multifunctional Viral Protein That Modulates Cellular Gene Expression and Functions. Oncotarget.

[25] Charnay N., Ivanyi-Nagy R., Soto-Rifo R., Ohlmann T., López-Lastra M., Darlix J.-L. (2009). Mechanism of HIV-1 Tat RNA Translation and Its Activation by the Tat Protein. Retrovirology.

[26] Alcamí J. (2008). Ciclo Replicativo Del VIH. Dianas Terapéuticas Consolidadas y Dianas Potenciales. Enferm. Infecc. Microbiol. Clin..

[27] Kirchhoff F. (2013). HIV Life Cycle: Overview. Encyclopedia of AIDS.

[28] Mann J.K., Byakwaga H., Kuang X.T., Le A.Q., Brumme C.J., Mwimanzi P., Omarjee S., Martin E., Lee G.Q., Baraki B. (2013). Ability of HIV-1 Nef to Downregulate CD4 and HLA Class I Differs among Viral Subtypes. Retrovirology.

[29] Malim M.H., Emerman M. (2008). HIV-1 Accessory Proteins—Ensuring Viral Survival in a Hostile Environment. Cell Host Microbe.

[30] Hokello J., Tyagi K., Owor R.O., Sharma A.L., Bhushan A., Daniel R., Tyagi M. (2024). New Insights into HIV Life Cycle, Th1/Th2 Shift during HIV Infection and Preferential Virus Infection of Th2 Cells: Implications of Early HIV Treatment Initiation and Care. Life.

[31] Patel K., Zhang A., Zhang M.H., Bunachita S., Baccouche B.M., Hundal H., Lavado L.K., Agarwal A., Malik P., Patel U.K. (2021). Forty Years Since the Epidemic: Modern Paradigms in HIV Diagnosis and Treatment. Cureus.

[32] McNeil R., Kerr T., Coleman B., Maher L., Milloy M.J., Small W. (2017). Antiretroviral Therapy Interruption among HIV Postive People Who Use Drugs in a Setting with a Community-Wide HIV Treatment-as-Prevention Initiative. AIDS Behav..

[33] Tsuda H., Koga M., Nojima M., Senkoji T., Kubota M., Kikuchi T., Adachi E., Ikeuchi K., Tsutsumi T., Koibuchi T. (2021). Changes in Survival and Causes of Death among People Living with HIV: Three Decades of Surveys from Tokyo, One of the Asian Metropolitan Cities. J. Infect. Chemother..

[34] Deeks S.G., Lewin S.R., Havlir D. (2013). V The End of AIDS: HIV Infection as a Chronic Disease. Lancet.

[35] Masters M.C., Krueger K.M., Williams J.L., Morrison L., Cohn S.E. (2019). Beyond One Pill, Once Daily: Current Challenges of Antiretroviral Therapy Management in the United States. Expert Rev. Clin. Pharmacol..

[36] WHO (2021). Consolidated Guidelines on HIV Prevention, Testing, Treatment, Service Delivery and Monitoring: Recommendations for a Public Health Approach.

[37] Pomerantz R.J., Horn D.L. (2003). Twenty Years of Therapy for HIV-1 Infection. Nat. Med..

[38] Pettit S.C., Moody M.D., Wehbie R.S., Kaplan A.H., Nantermet P.V., Klein C.A., Swanstrom R. (1994). The P2 Domain of Human Immunodeficiency Virus Type 1 Gag Regulates Sequential Proteolytic Processing and Is Required to Produce Fully Infectious Virions. J. Virol..

[39] Konvalinka J., Kräusslich H.G., Müller B. (2015). Retroviral Proteases and Their Roles in Virion Maturation. Virology.

[40] Oroszlan S., Luftig R.B. (1990). Retroviral Proteinases. Curr. Top. Microbiol. Immunol..

[41] Li P., Stephenson A.J., Brennan P.A., Karageorgos L., Kok T., Kuiper L.J., Swift L.J., Burrell C.J. (1994). Initiation of Reverse Transcription during Cell-to-Cell Transmission of Human Immunodeficiency Virus Infection Uses Pre-Existing Reverse Transcriptase. J. Gen. Virol..

[42] Chattopadhyay D., Evans D.B., Deibel M.R., Vosters A.F., Eckenrode F.M., Einspahr H.M., Hui J.O., Tomasselli A.G., Zurcher-Neely H.A., Heinrikson R.L. (1992). Purification and Characterization of Heterodimeric Human Immunodeficiency Virus Type 1 (HIV-1) Reverse Transcriptase Produced by in Vitro Processing of P66 with Recombinant HIV-1 Protease. J. Biol. Chem..

[43] Miller M.D., Farnet C.M., Bushman F.D. (1997). Human Immunodeficiency Virus Type 1 Preintegration Complexes: Studies of Organization and Composition. J. Virol..

[44] Fujiwara T., Mizuuchi K. (1988). Retroviral DNA Integration: Structure of an Integration Intermediate. Cell.

[45] Brown P.O., Bowerman B., Varmus H.E., Bishop J.M. (1989). Retroviral Integration: Structure of the Initial Covalent Product and Its Precursor, and a Role for the Viral IN Protein. Proc. Natl. Acad. Sci. USA.

[46] Engelman A., Mizuuchi K., Craigie R. (1991). HIV-1 DNA Integration: Mechanism of Viral DNA Cleavage and DNA Strand Transfer. Cell.

[47] Coffin J., Hughes S., Varmus H. (1997). The Interactions of Retroviruses and Their Hosts. Retroviruses.

[48] Engelman A., Cherepanov P. (2012). The Structural Biology of HIV-1: Mechanistic and Therapeutic Insights. Nat. Rev. Microbiol..

[49] Engelman A. (2003). The Roles of Cellular Factors in Retroviral Integration. Curr. Top. Microbiol. Immunol..

[50] Yoder K.E., Bushman F.D. (2000). Repair of Gaps in Retroviral DNA Integration Intermediates. J. Virol..

[51] Usach I., Melis V., Peris J.-E. (2013). Non-Nucleoside Reverse Transcriptase Inhibitors: A Review on Pharmacokinetics, Pharmacodynamics, Safety and Tolerability. J. Int. AIDS Soc..

[52] Rizza S.A., Bhatia R., Zeuli J., Temesgen Z. (2019). Ibalizumab for the Treatment of Multidrug-Resistant HIV-1 Infection. Drugs Today.

[53] Montessori V., Press N., Harris M., Akagi L., Montaner J.S.G. (2004). Adverse Effects of Antiretroviral Therapy for HIV Infection. CMAJ.

[54] Margolis A.M., Heverling H., Pham P.A., Stolbach A. (2014). A Review of the Toxicity of HIV Medications. J. Med. Toxicol..

[55] Eckhardt B.J., Gulick R.M. (2017). Drugs for HIV Infection. Infectious Diseases.

[56] European AIDS Clinical Society (2022). EACS Guidelines 2022.

[57] FDA Cabenuva (Cabotegravir Extended-Release Injectable Suspension; Rilpivirine Extended-Release Injectable Suspension), Co-Packaged for Intramuscular Use. https://www.accessdata.fda.gov/drugsatfda_docs/label/2021/212888s000lbl.pdf.

[58] Durham S.H., Chahine E.B. (2021). Cabotegravir-Rilpivirine: The First Complete Long-Acting Injectable Regimen for the Treatment of HIV-1 Infection. Ann. Pharmacother..

[59] Patel P.H., Zulfiqar H. (2015). Reverse Transcriptase Inhibitors. Frontiers in HIV Research.

[60] Bazzoli C., Jullien V., Le Tiec C., Rey E., Mentré F., Taburet A.M. (2010). Intracellular Pharmacokinetics of Antiretroviral Drugs in HIV-Infected Patients, and Their Correlation with Drug Action. Clin. Pharmacokinet..

[61] Gao W.Y., Shirasaka T., Johns D.G., Broder S., Mitsuya H. (1993). Differential Phosphorylation of Azidothymidine, Dideoxycytidine, and Dideoxyinosine in Resting and Activated Peripheral Blood Mononuclear Cells. J. Clin. Investig..

[62] Robbins B.L., Wilcox C.K., Fridland A., Rodman J.H. (2003). Metabolism of Tenofovir and Didanosine in Quiescent or Stimulated Human Peripheral Blood Mononuclear Cells. Pharmacotherapy.

[63] Arts E.J., Hazuda D.J. (2012). HIV-1 Antiretroviral Drug Therapy. Cold Spring Harb. Perspect. Med..

[64] De Clercq E. (2007). The Design of Drugs for HIV and HCV. Nat. Rev. Drug Discov..

[65] Martins S., Ramos M., Fernandes P. (2008). The Current Status of the NNRTI Family of Antiretrovirals Used Against HIV Infection. Curr. Med. Chem..

[66] Zhan P., Chen X., Li D., Fang Z., De Clercq E., Liu X. (2013). HIV-1 NNRTIs: Structural Diversity, Pharmacophore Similarity, and Impliations for Drug Design. Med. Res. Rev..

[67] Wang Y., De Clercq E., Li G. (2019). Current and Emerging Non-Nucleoside Reverse Transcriptase Inhibitors (NNRTIs) for HIV-1 Treatment. Expert Opin. Drug Metab. Toxicol..

[68] Zhuang C., Pannecouque C., De Clercq E., Chen F. (2020). Development of Non-Nucleoside Reverse Transcriptase Inhibitors (NNRTIs): Our Past Twenty Years. Acta Pharm. Sin. B.

[69] Das K., Martinez S.E., DeStefano J.J., Arnold E. (2019). Structure of HIV-1 RT/DsRNA Initiation Complex Prior to Nucleotide Incorporation. Proc. Natl. Acad. Sci. USA.

[70] Namasivayam V., Vanangamudi M., Kramer V.G., Kurup S., Zhan P., Liu X., Kongsted J., Byrareddy S.N. (2019). The Journey of HIV-1 Non-Nucleoside Reverse Transcriptase Inhibitors (NNRTIs) from Lab to Clinic. J. Med. Chem..

[71] National Institute of Diabetes and Digestive and Kidney Diseases (2012). LiverTox: Clinical and Research Information on Drug-Induced Liver Injury [Internet].

[72] Wang Y., Lv Z., Chu Y. (2015). HIV Protease Inhibitors: A Review of Molecular Selectivity and Toxicity. HIV/AIDS—Res. Palliat. Care.

[73] Li M., Oliveira Passos D., Shan Z., Smith S.J., Sun Q., Biswas A., Choudhuri I., Strutzenberg T.S., Haldane A., Deng N. (2023). Mechanisms of HIV-1 Integrase Resistance to Dolutegravir and Potent Inhibition of Drug-Resistant Variants. Sci. Adv..

[74] Delelis O., Carayon K., Saïb A., Deprez E., Mouscadet J.-F. (2008). Integrase and Integration: Biochemical Activities of HIV-1 Integrase. Retrovirology.

[75] Hazuda D.J., Felock P., Witmer M., Wolfe A., Stillmock K., Grobler J.A., Espeseth A., Gabryelski L., Schleif W., Blau C. (2000). Inhibitors of Strand Transfer That Prevent Integration and Inhibit HIV-1 Replication in Cells. Science.

[76] Irlbeck D.M., Amrine-Madsen H., Kitrinos K.M., LaBranche C.C., Demarest J.F. (2008). Chemokine (C-C Motif) Receptor 5-Using Envelopes Predominate in Dual/Mixed-Tropic HIV from the Plasma of Drug-Naive Individuals. AIDS.

[77] Mosier D.E. (2008). How HIV Changes Its Tropism: Evolution and Adaptation?. Curr. Opin. HIV AIDS.

[78] Vandekerckhove L., Verhofstede C., Vogelaers D. (2009). Maraviroc: Perspectives for Use in Antiretroviral-Naive HIV-1-Infected Patients. J. Antimicrob. Chemother..

[79] Gulick R.M., Fatkenheuer G., Burnside R., Hardy W.D., Nelson M.R., Goodrich J., Mukwaya G., Portsmouth S., Heera J.R. (2014). Five-Year Safety Evaluation of Maraviroc in HIV-1–Infected Treatment-Experienced Patients. JAIDS J. Acquir. Immune Defic. Syndr..

[80] Saag M., Goodrich J., Fätkenheuer G., Clotet B., Clumeck N., Sullivan J., Westby M., van der Ryst E., Mayer H. (2009). A Double-Blind, Placebo-Controlled Trial of Maraviroc in Treatment-Experienced Patients Infected with Non-R5 HIV-1. J. Infect. Dis..

[81] Tan Q., Zhu Y., Li J., Chen Z., Han G.W., Kufareva I., Li T., Ma L., Fenalti G., Li J. (2013). Structure of the CCR5 Chemokine Receptor–HIV Entry Inhibitor Maraviroc Complex. Science.

[82] Seval N., Frank C., Kozal M. (2021). Fostemsavir for the Treatment of HIV. Expert Rev. Anti. Infect. Ther..

[83] Kozal M., Aberg J., Pialoux G., Cahn P., Thompson M., Molina J.-M., Grinsztejn B., Diaz R., Castagna A., Kumar P. (2020). Fostemsavir in Adults with Multidrug-Resistant HIV-1 Infection. N. Engl. J. Med..

[84] Meanwell N.A., Krystal M.R., Nowicka-Sans B., Langley D.R., Conlon D.A., Eastgate M.D., Grasela D.M., Timmins P., Wang T., Kadow J.F. (2018). Inhibitors of HIV-1 Attachment: The Discovery and Development of Temsavir and Its Prodrug Fostemsavir. J. Med. Chem..

[85] Pancera M., Lai Y.-T., Bylund T., Druz A., Narpala S., O’Dell S., Schön A., Bailer R.T., Chuang G.-Y., Geng H. (2017). Crystal Structures of Trimeric HIV Envelope with Entry Inhibitors BMS-378806 and BMS-626529. Nat. Chem. Biol..

[86] Lalezari J.P., Henry K., O’Hearn M., Montaner J.S.G., Piliero P.J., Trottier B., Walmsley S., Cohen C., Kuritzkes D.R., Eron J.J. (2003). Enfuvirtide, an HIV-1 Fusion Inhibitor, for Drug-Resistant HIV Infection in North and South America. N. Engl. J. Med..

[87] Matthews T., Salgo M., Greenberg M., Chung J., DeMasi R., Bolognesi D. (2004). Enfuvirtide: The First Therapy to Inhibit the Entry of HIV-1 into Host CD4 Lymphocytes. Nat. Rev. Drug Discov..

[88] Lazzarin A. (2005). Enfuvirtide: The First HIV Fusion Inhibitor. Expert Opin. Pharmacother..

[89] Jamjian M.C., McNicholl I.R. (2004). Enfuvirtide: First Fusion Inhibitor for Treatment of HIV Infection. Am. J. Health-Syst. Pharm..

[90] Follis K.E., Larson S.J., Lu M., Nunberg J.H. (2002). Genetic Evidence That Interhelical Packing Interactions in the Gp41 Core Are Critical for Transition of the Human Immunodeficiency Virus Type 1 Envelope Glycoprotein to the Fusion-Active State. J. Virol..

[91] Beccari M.V., Mogle B.T., Sidman E.F., Mastro K.A., Asiago-Reddy E., Kufel W.D. (2019). Ibalizumab, a Novel Monoclonal Antibody for the Management of Multidrug-Resistant HIV-1 Infection. Antimicrob. Agents Chemother..

[92] Bettiker R.L., Koren D.E., Jacobson J.M. (2018). Ibalizumab. Curr. Opin. HIV AIDS.

[93] Pace C.S., Fordyce M.W., Franco D., Kao C.-Y., Seaman M.S., Ho D.D. (2013). Anti-CD4 Monoclonal Antibody Ibalizumab Exhibits Breadth and Potency Against HIV-1, with Natural Resistance Mediated by the Loss of a V5 Glycan in Envelope. JAIDS J. Acquir. Immune Defic. Syndr..

[94] Song R., Franco D., Kao C.-Y., Yu F., Huang Y., Ho D.D. (2010). Epitope Mapping of Ibalizumab, a Humanized Anti-CD4 Monoclonal Antibody with Anti-HIV-1 Activity in Infected Patients. J. Virol..

[95] Freeman M.M., Seaman M.S., Rits-Volloch S., Hong X., Kao C.-Y., Ho D.D., Chen B. (2010). Crystal Structure of HIV-1 Primary Receptor CD4 in Complex with a Potent Antiviral Antibody. Structure.

[96] Iacob S.A., Iacob D.G. (2017). Ibalizumab Targeting CD4 Receptors, An Emerging Molecule in HIV Therapy. Front. Microbiol..

[97] Gulick R.M., Flexner C. (2019). Long-Acting HIV Drugs for Treatment and Prevention. Annu. Rev. Med..

[98] Gendelman H.E., McMillan J., Bade A.N., Edagwa B., Kevadiya B.D. (2019). The Promise of Long-Acting Antiretroviral Therapies: From Need to Manufacture. Trends Microbiol..

[99] Pinto R.M., Hall E., Tomlin R. (2023). Injectable Long-Acting Cabotegravir–Rilpivirine Therapy for People Living With HIV/AIDS: Addressing Implementation Barriers From the Start. J. Assoc. Nurses AIDS Care.

[100] Slama L., Porcher R., Linard F., Chakvetadze C., Cros A., Carillon S., Gallardo L., Viard J.-P., Molina J.-M. (2023). Injectable Long Acting Antiretroviral for HIV Treatment and Prevention: Perspectives of Potential Users. BMC Infect. Dis..

[101] Moreno S., Rivero A., Ventayol P., Falcó V., Torralba M., Schroeder M., Neches V., Vallejo-Aparicio L.A., Mackenzie I., Turner M. (2023). Cabotegravir and Rilpivirine Long-Acting Antiretroviral Therapy Administered Every 2 Months Is Cost-Effective for the Treatment of HIV-1 in Spain. Infect. Dis. Ther..

[102] Bares S.H., Scarsi K.K. (2022). A New Paradigm for Antiretroviral Delivery: Long-Acting Cabotegravir and Rilpivirine for the Treatment and Prevention of HIV. Curr. Opin. HIV AIDS.

[103] Thoueille P., Choong E., Cavassini M., Buclin T., Decosterd L.A. (2022). Long-Acting Antiretrovirals: A New Era for the Management and Prevention of HIV Infection. J. Antimicrob. Chemother..

[104] Cobb D.A., Smith N.A., Edagwa B.J., McMillan J.M. (2020). Long-Acting Approaches for Delivery of Antiretroviral Drugs for Prevention and Treatment of HIV: A Review of Recent Research. Expert Opin. Drug Deliv..

[105] UNAIDS Global HIV & AIDS Statistics—Fact Sheet. 2023. https://www.unaids.org/sites/default/files/media_asset/UNAIDS_FactSheet_en.pdf.

[106] WHO (2021). HIV Drug Resistance Report 2021.

[107] Pennings P.S. (2013). HIV Drug Resistance: Problems and Perspectives. Infect. Dis. Rep..

[108] Zdanowicz M.M. (2006). The Pharmacology of HIV Drug Resistance. Am. J. Pharm. Educ..

[109] Richman D.D., Morton S.C., Wrin T., Hellmann N., Berry S., Shapiro M.F., Bozzette S.A. (2004). The Prevalence of Antiretroviral Drug Resistance in the United States. AIDS.

[110] Frentz D., van de Vijver D., Abecasis A., Albert J., Hamouda O., Jørgensen L., Kücherer C., Struck D., Schmit J.-C., Vercauteren J. (2014). Patterns of Transmitted HIV Drug Resistance in Europe Vary by Risk Group. PLoS ONE.

[111] Yerly S., Vora S., Rizzardi P., Chave J.-P., Vernazza P.L., Flepp M., Telenti A., Battegay M., Veuthey A.-L., Bru J.-P. (2001). Acute HIV Infection: Impact on the Spread of HIV and Transmission of Drug Resistance. AIDS.

[112] Pao D., Fisher M., Hué S., Dean G., Murphy G., Cane P.A., Sabin C.A., Pillay D. (2005). Transmission of HIV-1 during Primary Infection: Relationship to Sexual Risk and Sexually Transmitted Infections. AIDS.

[113] Brenner B.G., Roger M., Routy J., Moisi D., Ntemgwa M., Matte C., Baril J., Thomas R., Rouleau D., Bruneau J. (2007). High Rates of Forward Transmission Events after Acute/Early HIV-1 Infection. J. Infect. Dis..

[114] Temereanca A., Ruta S. (2023). Strategies to Overcome HIV Drug Resistance-Current and Future Perspectives. Front. Microbiol..

[115] Kühnert D., Kouyos R., Shirreff G., Pečerska J., Scherrer A.U., Böni J., Yerly S., Klimkait T., Aubert V., Günthard H.F. (2018). Quantifying the Fitness Cost of HIV-1 Drug Resistance Mutations through Phylodynamics. PLoS Pathog..

[116] Wittkop L., Günthard H.F., de Wolf F., Dunn D., Cozzi-Lepri A., de Luca A., Kücherer C., Obel N., von Wyl V., Masquelier B. (2011). Effect of Transmitted Drug Resistance on Virological and Immunological Response to Initial Combination Antiretroviral Therapy for HIV (EuroCoord-CHAIN Joint Project): A European Multicohort Study. Lancet Infect. Dis..

[117] Gupta R.K., Jordan M.R., Sultan B.J., Hill A., Davis D.H., Gregson J., Sawyer A.W., Hamers R.L., Ndembi N., Pillay D. (2012). Global Trends in Antiretroviral Resistance in Treatment-Naive Individuals with HIV after Rollout of Antiretroviral Treatment in Resource-Limited Settings: A Global Collaborative Study and Meta-Regression Analysis. Lancet.

[118] Gupta R.K., Gregson J., Parkin N., Haile-Selassie H., Tanuri A., Andrade Forero L., Kaleebu P., Watera C., Aghokeng A., Mutenda N. (2018). HIV-1 Drug Resistance before Initiation or Re-Initiation of First-Line Antiretroviral Therapy in Low-Income and Middle-Income Countries: A Systematic Review and Meta-Regression Analysis. Lancet Infect. Dis..

[119] Miranda M.N.S., Pingarilho M., Pimentel V., Martins M.d.R.O., Kaiser R., Seguin-Devaux C., Paredes R., Zazzi M., Incardona F., Abecasis A.B. (2022). Trends of Transmitted and Acquired Drug Resistance in Europe from 1981 to 2019: A Comparison between the Populations of Late Presenters and Non-Late Presenters. Front. Microbiol..

[120] Stadeli K.M., Richman D.D. (2013). Rates of Emergence of HIV Drug Resistance in Resource-Limited Settings: A Systematic Review. Antivir. Ther..

[121] Hauser A., Goldstein F., Reichmuth M.L., Kouyos R.D., Wandeler G., Egger M., Riou J. (2022). Acquired HIV Drug Resistance Mutations on First-Line Antiretroviral Therapy in Southern Africa: Systematic Review and Bayesian Evidence Synthesis. J. Clin. Epidemiol..

[122] Shirasaka T., Kavlick M.F., Ueno T., Gao W.Y., Kojima E., Alcaide M.L., Chokekijchai S., Roy B.M., Arnold E., Yarchoan R. (1995). Emergence of Human Immunodeficiency Virus Type 1 Variants with Resistance to Multiple Dideoxynucleosides in Patients Receiving Therapy with Dideoxynucleosides. Proc. Natl. Acad. Sci. USA.

[123] Marcelin A.-G. (2006). Resistance to Nucleoside Reverse Transcriptase Inhibitors. Antiretroviral Resistance in Clinical Practice.

[124] Winters M.A., Coolley K.L., Girard Y.A., Levee D.J., Hamdan H., Shafer R.W., Katzenstein D.A., Merigan T.C. (1998). A 6-Basepair Insert in the Reverse Transcriptase Gene of Human Immunodeficiency Virus Type 1 Confers Resistance to Multiple Nucleoside Inhibitors. J. Clin. Investig..

[125] de Jong J.J., Goudsmit J., Lukashov V.V., Hillebrand M.E., Baan E., Huismans R., Danner S.A., ten Veen J.H., de Wolf F., Jurriaans S. (1999). Insertion of Two Animo Acids Combined with Changes in Reverse Transcriptase Containing Tyrosine-215 of HIV-1 Resistant to Multiple Nucleoside Analogs. AIDS.

[126] Richman D.D. (1996). Antiretroviral Drug Resistance: Mechanisms, Pathogenesis, Clinical Significance. Antiviral Chemotherapy 4. Advances in Experimental Medicine and Biology.

[127] Shafer R.W., Schapiro J.M. (2008). HIV-1 Drug Resistance Mutations: An Updated Framework for the Second Decade of HAART. AIDS Rev..

[128] Brenner B., Turner D., Oliveira M., Moisi D., Detorio M., Carobene M., Marlink R.G., Schapiro J., Roger M., Wainberg M.A. (2003). A V106M Mutation in HIV-1 Clade C Viruses Exposed to Efavirenz Confers Cross-Resistance to Non-Nucleoside Reverse Transcriptase Inhibitors. AIDS.

[129] Sarafianos S.G., Das K., Hughes S.H., Arnold E. (2004). Taking Aim at a Moving Target: Designing Drugs to Inhibit Drug-Resistant HIV-1 Reverse Transcriptases. Curr. Opin. Struct. Biol..

[130] Ren J., Stammers D.K. (2008). Structural Basis for Drug Resistance Mechanisms for Non-Nucleoside Inhibitors of HIV Reverse Transcriptase. Virus Res..

[131] Lecossier D., Shulman N.S., Morand-Joubert L., Shafer R.W., Joly V., Zolopa A.R., Clavel F., Hance A.J. (2005). Detection of Minority Populations of HIV-1 Expressing the K103N Resistance Mutation in Patients Failing Nevirapine. JAIDS J. Acquir. Immune Defic. Syndr..

[132] Rhee S.-Y., Taylor J., Wadhera G., Ben-Hur A., Brutlag D.L., Shafer R.W. (2006). Genotypic Predictors of Human Immunodeficiency Virus Type 1 Drug Resistance. Proc. Natl. Acad. Sci. USA.

[133] Bacheler L., Jeffrey S., Hanna G., D’Aquila R., Wallace L., Logue K., Cordova B., Hertogs K., Larder B., Buckery R. (2001). Genotypic Correlates of Phenotypic Resistance to Efavirenz in Virus Isolates from Patients Failing Nonnucleoside Reverse Transcriptase Inhibitor Therapy. J. Virol..

[134] Vingerhoets J., Azijn H., Fransen E., De Baere I., Smeulders L., Jochmans D., Andries K., Pauwels R., de Béthune M.-P. (2005). TMC125 Displays a High Genetic Barrier to the Development of Resistance: Evidence from In Vitro Selection Experiments. J. Virol..

[135] Parkin N.T., Gupta S., Chappey C., Petropoulos C.J. (2006). The K101P and K103R/V179D Mutations in Human Immunodeficiency Virus Type 1 Reverse Transcriptase Confer Resistance to Nonnucleoside Reverse Transcriptase Inhibitors. Antimicrob. Agents Chemother..

[136] Rhee S.-Y. (2003). Human Immunodeficiency Virus Reverse Transcriptase and Protease Sequence Database. Nucleic Acids Res..

[137] Brown A.J.L., Precious H.M., Whitcomb J.M., Wong J.K., Quigg M., Huang W., Daar E.S., D’Aquila R.T., Keiser P.H., Connick E. (2000). Reduced Susceptibility of Human Immunodeficiency Virus Type 1 (HIV-1) from Patients with Primary HIV Infection to Nonnucleoside Reverse Transcriptase Inhibitors Is Associated with Variation at Novel Amino Acid Sites. J. Virol..

[138] Ceccherini-Silberstein F., Svicher V., Sing T., Artese A., Santoro M.M., Forbici F., Bertoli A., Alcaro S., Palamara G., d’Arminio Monforte A. (2007). Characterization and Structural Analysis of Novel Mutations in Human Immunodeficiency Virus Type 1 Reverse Transcriptase Involved in the Regulation of Resistance to Nonnucleoside Inhibitors. J. Virol..

[139] Ghosh A.K., Chapsal B.D., Weber I.T., Mitsuya H. (2008). Design of HIV Protease Inhibitors Targeting Protein Backbone: An Effective Strategy for Combating Drug Resistance. Acc. Chem. Res..

[140] Wensing A.M.J., van Maarseveen N.M., Nijhuis M. (2010). Fifteen Years of HIV Protease Inhibitors: Raising the Barrier to Resistance. Antivir. Res..

[141] Johnson V.A., Brun-Vezinet F., Clotet B., Gunthard H.F., Kuritzkes D.R., Pillay D., Schapiro J.M., Richman D.D. (2008). Update of the Drug Resistance Mutations in HIV-1. Top. HIV Med..

[142] Cooper D.A., Steigbigel R.T., Gatell J.M., Rockstroh J.K., Katlama C., Yeni P., Lazzarin A., Clotet B., Kumar P.N., Eron J.E. (2008). Subgroup and Resistance Analyses of Raltegravir for Resistant HIV-1 Infection. N. Engl. J. Med..

[143] Kobayashi M., Nakahara K., Seki T., Miki S., Kawauchi S., Suyama A., Wakasamorimoto C., Kodama M., Endoh T., Oosugi E. (2008). Selection of Diverse and Clinically Relevant Integrase Inhibitor-Resistant Human Immunodeficiency Virus Type 1 Mutants. Antivir. Res..

[144] Steigbigel R.T., Cooper D.A., Teppler H., Eron J.J., Gatell J.M., Kumar P.N., Rockstroh J.K., Schechter M., Katlama C., Markowitz M. (2010). Long-Term Efficacy and Safety of Raltegravir Combined with Optimized Background Therapy in Treatment-Experienced Patients with Drug-Resistant HIV Infection: Week 96 Results of the BENCHMRK 1 and 2 Phase III Trials. Clin. Infect. Dis..

[145] Van Wesenbeeck L., Rondelez E., Feyaerts M., Verheyen A., Van der Borght K., Smits V., Cleybergh C., De Wolf H., Van Baelen K., Stuyver L.J. (2011). Cross-Resistance Profile Determination of Two Second-Generation HIV-1 Integrase Inhibitors Using a Panel of Recombinant Viruses Derived from Raltegravir-Treated Clinical Isolates. Antimicrob. Agents Chemother..

[146] Bar-Magen T., Sloan R.D., Faltenbacher V.H., Donahue D.A., Kuhl B.D., Oliveira M., Xu H., Wainberg M.A. (2009). Comparative Biochemical Analysis of HIV-1 Subtype B and C Integrase Enzymes. Retrovirology.

[147] Bar-Magen T., Sloan R.D., Donahue D.A., Kuhl B.D., Zabeida A., Xu H., Oliveira M., Hazuda D.J., Wainberg M.A. (2010). Identification of Novel Mutations Responsible for Resistance to MK-2048, a Second-Generation HIV-1 Integrase Inhibitor. J. Virol..

[148] Li X., Krishnan L., Cherepanov P., Engelman A. (2011). Structural Biology of Retroviral DNA Integration. Virology.

[149] Bacchi A., Carcelli M., Compari C., Fisicaro E., Pala N., Rispoli G., Rogolino D., Sanchez T.W., Sechi M., Sinisi V. (2011). Investigating the Role of Metal Chelation in HIV-1 Integrase Strand Transfer Inhibitors. J. Med. Chem..

[150] Hare S., Smith S.J., Métifiot M., Jaxa-Chamiec A., Pommier Y., Hughes S.H., Cherepanov P. (2011). Structural and Functional Analyses of the Second-Generation Integrase Strand Transfer Inhibitor Dolutegravir (S/GSK1349572). Mol. Pharmacol..

[151] Hare S., Vos A.M., Clayton R.F., Thuring J.W., Cummings M.D., Cherepanov P. (2010). Molecular Mechanisms of Retroviral Integrase Inhibition and the Evolution of Viral Resistance. Proc. Natl. Acad. Sci. USA.

[152] Hare S., Gupta S.S., Valkov E., Engelman A., Cherepanov P. (2010). Retroviral Intasome Assembly and Inhibition of DNA Strand Transfer. Nature.

[153] Espeseth A.S., Felock P., Wolfe A., Witmer M., Grobler J., Anthony N., Egbertson M., Melamed J.Y., Young S., Hamill T. (2000). HIV-1 Integrase Inhibitors That Compete with the Target DNA Substrate Define a Unique Strand Transfer Conformation for Integrase. Proc. Natl. Acad. Sci. USA.

[154] Mesplède T., Quashie P.K., Wainberg M.A. (2012). Resistance to HIV Integrase Inhibitors. Curr. Opin. HIV AIDS.

[155] Mbhele N., Chimukangara B., Gordon M. (2021). HIV-1 Integrase Strand Transfer Inhibitors: A Review of Current Drugs, Recent Advances and Drug Resistance. Int. J. Antimicrob. Agents.

[156] Temesgen Z. (2008). Raltegravir: First in Class HIV Integrase Inhibitor. Ther. Clin. Risk Manag..

[157] Wohl D.A., Cohen C., Gallant J.E., Mills A., Sax P.E., DeJesus E., Zolopa A., Liu H.C., Plummer A., White K.L. (2014). A Randomized, Double-Blind Comparison of Single-Tablet Regimen Elvitegravir/Cobicistat/Emtricitabine/Tenofovir DF Versus Single-Tablet Regimen Efavirenz/Emtricitabine/Tenofovir DF for Initial Treatment of HIV-1 Infection. JAIDS J. Acquir. Immune Defic. Syndr..

[158] Clumeck N., Molina J.-M., Henry K., Gathe J., Rockstroh J.K., DeJesus E., Wei X., White K., Fordyce M.W., Rhee M.S. (2014). A Randomized, Double-Blind Comparison of Single-Tablet Regimen Elvitegravir/Cobicistat/Emtricitabine/Tenofovir DF vs Ritonavir-Boosted Atazanavir Plus Emtricitabine/Tenofovir DF for Initial Treatment of HIV-1 Infection. JAIDS J. Acquir. Immune Defic. Syndr..

[159] Lübke N., Jensen B., Hüttig F., Feldt T., Walker A., Thielen A., Däumer M., Obermeier M., Kaiser R., Knops E. (2019). Failure of Dolutegravir First-Line ART with Selection of Virus Carrying R263K and G118R. N. Engl. J. Med..

[160] Wensing A.M., Calvez V., Ceccherini-Silberstein F., Charpentier C., Günthard H.F., Paredes R., Shafer R.W., Richman D.D. (2019). 2019 Update of the Drug Resistance Mutations in HIV-1. Top. Antivir. Med..

[161] Labrosse B., Labernardière J.-L., Dam E., Trouplin V., Skrabal K., Clavel F., Mammano F. (2003). Baseline Susceptibility of Primary Human Immunodeficiency Virus Type 1 to Entry Inhibitors. J. Virol..

[162] Reeves J.D., Gallo S.A., Ahmad N., Miamidian J.L., Harvey P.E., Sharron M., Pöhlmann S., Sfakianos J.N., Derdeyn C.A., Blumenthal R. (2002). Sensitivity of HIV-1 to Entry Inhibitors Correlates with Envelope/Coreceptor Affinity, Receptor Density, and Fusion Kinetics. Proc. Natl. Acad. Sci. USA.

[163] Reeves J.D., Miamidian J.L., Biscone M.J., Lee F.-H., Ahmad N., Pierson T.C., Doms R.W. (2004). Impact of Mutations in the Coreceptor Binding Site on Human Immunodeficiency Virus Type 1 Fusion, Infection, and Entry Inhibitor Sensitivity. J. Virol..

[164] Ray N. (2008). Maraviroc in the Treatment of HIV Infection. Drug Des. Dev. Ther..

[165] Poveda E., Rodés B., Toro C., Martín-Carbonero L., Gonzalez-Lahoz J., Soriano V. (2002). Evolution of the Gp41 Env Region in HIV-Infected Patients Receiving T-20, a Fusion Inhibitor. AIDS.

[166] Rimsky L.T., Shugars D.C., Matthews T.J. (1998). Determinants of Human Immunodeficiency Virus Type 1 Resistance to Gp41-Derived Inhibitory Peptides. J. Virol..

[167] Wei X., Decker J.M., Liu H., Zhang Z., Arani R.B., Kilby J.M., Saag M.S., Wu X., Shaw G.M., Kappes J.C. (2002). Emergence of Resistant Human Immunodeficiency Virus Type 1 in Patients Receiving Fusion Inhibitor (T-20) Monotherapy. Antimicrob. Agents Chemother..

[168] Mink M., Mosier S.M., Janumpalli S., Davison D., Jin L., Melby T., Sista P., Erickson J., Lambert D., Stanfield-Oakley S.A. (2005). Impact of Human Immunodeficiency Virus Type 1 Gp41 Amino Acid Substitutions Selected during Enfuvirtide Treatment on Gp41 Binding and Antiviral Potency of Enfuvirtide In Vitro. J. Virol..

[169] Lu J., Deeks S.G., Hoh R., Beatty G., Kuritzkes B.A., Martin J.N., Kuritzkes D.R. (2006). Rapid Emergence of Enfuvirtide Resistance in HIV-1-Infected Patients. JAIDS J. Acquir. Immune Defic. Syndr..

[170] Xu L., Pozniak A., Wildfire A., Stanfield-Oakley S.A., Mosier S.M., Ratcliffe D., Workman J., Joall A., Myers R., Smit E. (2005). Emergence and Evolution of Enfuvirtide Resistance Following Long-Term Therapy Involves Heptad Repeat 2 Mutations within Gp41. Antimicrob. Agents Chemother..

[171] Greenberg M.L. (2004). Resistance to Enfuvirtide, the First HIV Fusion Inhibitor. J. Antimicrob. Chemother..

[172] Leung P.H.M., Chen J.H.K., Wong K.H., Chan K.C., Lam H.Y., Cheng V.C.C., Yuen K.Y., Yam W.C. (2010). High Prevalence of Primary Enfuvirtide (ENF) Resistance-Associated Mutations in HIV-1-Infected Patients in Hong Kong. J. Clin. Virol..

[173] Hughes C.A., Robinson L., Tseng A., MacArthur R.D. (2009). New Antiretroviral Drugs: A Review of the Efficacy, Safety, Pharmacokinetics, and Resistance Profile of Tipranavir, Darunavir, Etravirine, Rilpivirine, Maraviroc, and Raltegravir. Expert Opin. Pharmacother..

[174] Pugach P., Marozsan A.J., Ketas T.J., Landes E.L., Moore J.P., Kuhmann S.E. (2007). HIV-1 Clones Resistant to a Small Molecule CCR5 Inhibitor Use the Inhibitor-Bound Form of CCR5 for Entry. Virology.

[175] Roche M., Salimi H., Duncan R., Wilkinson B.L., Chikere K., Moore M.S., Webb N.E., Zappi H., Sterjovski J., Flynn J.K. (2013). A Common Mechanism of Clinical HIV-1 Resistance to the CCR5 Antagonist Maraviroc despite Divergent Resistance Levels and Lack of Common Gp120 Resistance Mutations. Retrovirology.

[176] Trkola A., Kuhmann S.E., Strizki J.M., Maxwell E., Ketas T., Morgan T., Pugach P., Xu S., Wojcik L., Tagat J. (2002). HIV-1 Escape from a Small Molecule, CCR5-Specific Entry Inhibitor Does Not Involve CXCR4 Use. Proc. Natl. Acad. Sci. USA.

[177] Hiryak K., Koren D.E. (2021). Fostemsavir: A Novel Attachment Inhibitor for Patients With Multidrug-Resistant HIV-1 Infection. Ann. Pharmacother..

[178] Berruti M., Pincino R., Taramasso L., Di Biagio A. (2021). Evaluating Fostemsavir as a Therapeutic Option for Patients with HIV. Expert Opin. Pharmacother..

[179] Zhou N., Nowicka-Sans B., McAuliffe B., Ray N., Eggers B., Fang H., Fan L., Healy M., Langley D.R., Hwang C. (2014). Genotypic Correlates of Susceptibility to HIV-1 Attachment Inhibitor BMS-626529, the Active Agent of the Prodrug BMS-663068. J. Antimicrob. Chemother..

[180] Lataillade M., Zhou N., Joshi S.R., Lee S., Stock D.A., Hanna G.J., Krystal M. (2018). Viral Drug Resistance Through 48 Weeks, in a Phase 2b, Randomized, Controlled Trial of the HIV-1 Attachment Inhibitor Prodrug, Fostemsavir. JAIDS J. Acquir. Immune Defic. Syndr..

[181] Lepore L., Fabrizio C., Bavaro D.F., Milano E., Volpe A., Lagioia A., Angarano G., Saracino A., Monno L. (2020). Gp120 Substitutions at Positions Associated with Resistance to Fostemsavir in Treatment-Naive HIV-1-Positive Individuals. J. Antimicrob. Chemother..

[182] Alessandri-Gradt E., Charpentier C., Leoz M., Mourez T., Descamps D., Plantier J.-C. (2018). Impact of Natural Polymorphisms of HIV-1 Non-Group M on Genotypic Susceptibility to the Attachment Inhibitor Fostemsavir. J. Antimicrob. Chemother..

[183] Madani N., Perdigoto A.L., Srinivasan K., Cox J.M., Chruma J.J., LaLonde J., Head M., Smith A.B., Sodroski J.G. (2004). Localized Changes in the Gp120 Envelope Glycoprotein Confer Resistance to Human Immunodeficiency Virus Entry Inhibitors BMS-806 and #155. J. Virol..

[184] Nowicka-Sans B., Gong Y.-F., McAuliffe B., Dicker I., Ho H.-T., Zhou N., Eggers B., Lin P.-F., Ray N., Wind-Rotolo M. (2012). In Vitro Antiviral Characteristics of HIV-1 Attachment Inhibitor BMS-626529, the Active Component of the Prodrug BMS-663068. Antimicrob. Agents Chemother..

[185] Ray N., Hwang C., Healy M.D., Whitcomb J., Lataillade M., Wind-Rotolo M., Krystal M., Hanna G.J. (2013). Prediction of Virological Response and Assessment of Resistance Emergence to the HIV-1 Attachment Inhibitor BMS-626529 During 8-Day Monotherapy With Its Prodrug BMS-663068. JAIDS J. Acquir. Immune Defic. Syndr..

[186] Soulie C., Lambert-Niclot S., Fofana D.B., Fourati S., Ait-Arkoub Z., Sayon S., Simon A., Katlama C., Calvez V., Marcelin A.-G. (2013). Frequency of Amino Acid Changes Associated with Resistance to Attachment Inhibitor BMS-626529 in R5- and X4-Tropic HIV-1 Subtype B. J. Antimicrob. Chemother..

[187] Fofana D.B., Charpentier C., Maiga A.I., Lambert-Niclot S., Sayon S., Desire N., Simon A., Yazdanpanah Y., Katlama C., Descamps D. (2015). Genetic Barrier for Attachment Inhibitor BMS-626529 Resistance in HIV-1 B and Non-B Subtypes. J. Antimicrob. Chemother..

[188] Ballana E., Esté J.A. (2015). BMS-663068, a Safe and Effective HIV-1 Attachment Inhibitor. Lancet HIV.

[189] Charpentier C., Larrouy L., Visseaux B., Landman R., Levittas M., Storto A., Damond F., Yazdanpanah Y., Yeni P., Brun-Vezinet F. (2012). Prevalence of Subtype-Related Polymorphisms Associated with in Vitro Resistance to Attachment Inhibitor BMS-626529 in HIV-1 ‘Non-B’-Infected Patients. J. Antimicrob. Chemother..

[190] Li Z., Zhou N., Sun Y., Ray N., Lataillade M., Hanna G.J., Krystal M. (2013). Activity of the HIV-1 Attachment Inhibitor BMS-626529, the Active Component of the Prodrug BMS-663068, against CD4-Independent Viruses and HIV-1 Envelopes Resistant to Other Entry Inhibitors. Antimicrob. Agents Chemother..

[191] Rose R., Gartland M., Li Z., Zhou N., Cockett M., Beloor J., Lataillade M., Ackerman P., Krystal M. (2022). Clinical Evidence for a Lack of Cross-Resistance between Temsavir and Ibalizumab or Maraviroc. AIDS.

[192] Bouba Y., Berno G., Fabeni L., Carioti L., Salpini R., Aquaro S., Svicher V., Perno C.F., Ceccherini-Silberstein F., Santoro M.M. (2020). Identification of Gp120 Polymorphisms in HIV-1 B Subtype Potentially Associated with Resistance to Fostemsavir. J. Antimicrob. Chemother..

[193] Toma J., Weinheimer S.P., Stawiski E., Whitcomb J.M., Lewis S.T., Petropoulos C.J., Huang W. (2011). Loss of Asparagine-Linked Glycosylation Sites in Variable Region 5 of Human Immunodeficiency Virus Type 1 Envelope Is Associated with Resistance to CD4 Antibody Ibalizumab. J. Virol..

[194] Fessel W.J., Anderson B., Follansbee S.E., Winters M.A., Lewis S.T., Weinheimer S.P., Petropoulos C.J., Shafer R.W. (2011). The Efficacy of an Anti-CD4 Monoclonal Antibody for HIV-1 Treatment. Antivir. Res..

[195] De Feo C., Weiss C. (2012). Escape from Human Immunodeficiency Virus Type 1 (HIV-1) Entry Inhibitors. Viruses.

[196] Steegen K., Chandiwana N., Sokhela S., Venter W.D.F., Hans L. (2023). Impact of Rilpivirine Cross-Resistance on Long-Acting Cabotegravir-Rilpivirine in Low and Middle-Income Countries. AIDS.

[197] van Welzen B.J., Van Lelyveld S.F.L., Ter Beest G., Gisolf J.H., Geerlings S.E., Prins J.M., Van Twillert G., Van Nieuwkoop C., Van der Valk M., Burger D. (2024). Virological Failure after Switch to Long-Acting Cabotegravir and Rilpivirine Injectable Therapy: An In-Depth Analysis. Clin. Infect. Dis..

[198] Cervo A., Russo A., Di Carlo D., De Vito A., Fabeni L., D’Anna S., Duca L., Colpani A., Fois M., Zauli B. (2023). Long-Acting Combination of Cabotegravir plus Rilpivirine: A Picture of Potential Eligible and Ineligible HIV-Positive Individuals from the Italian ARCA Cohort. J. Glob. Antimicrob. Resist..

[199] Iyidogan P., Anderson K. (2014). Current Perspectives on HIV-1 Antiretroviral Drug Resistance. Viruses.

[200] Liu X., Patil S., Guo X., Wen F., Zhang X., Zhong Z., Wang X. (2024). Clinical, Epidemiological, and Drug Resistance Insights into HIV-Positive Patients in Meizhou, China. Front. Cell. Infect. Microbiol..

[201] Su C.T.-T., Ling W.-L., Lua W.-H., Haw Y.-X., Gan S.K.-E. (2016). Structural Analyses of 2015-Updated Drug-Resistant Mutations in HIV-1 Protease: An Implication of Protease Inhibitor Cross-Resistance. BMC Bioinform..

[202] Rhee S.-Y., Taylor J., Fessel W.J., Kaufman D., Towner W., Troia P., Ruane P., Hellinger J., Shirvani V., Zolopa A. (2010). HIV-1 Protease Mutations and Protease Inhibitor Cross-Resistance. Antimicrob. Agents Chemother..

[203] Jacobson J.M., Kuritzkes D.R., Godofsky E., DeJesus E., Larson J.A., Weinheimer S.P., Lewis S.T. (2009). Safety, Pharmacokinetics, and Antiretroviral Activity of Multiple Doses of Ibalizumab (Formerly TNX-355), an Anti-CD4 Monoclonal Antibody, in Human Immunodeficiency Virus Type 1-Infected Adults. Antimicrob. Agents Chemother..

[204] Blair H.A. (2020). Ibalizumab: A Review in Multidrug-Resistant HIV-1 Infection. Drugs.

[205] Grover D., Copas A., Green H., Edwards S.G., Dunn D.T., Sabin C., Phillips A., Allen E., Pillay D. (2008). What Is the Risk of Mortality Following Diagnosis of Multidrug-Resistant HIV-1?. J. Antimicrob. Chemother..

[206] Osterberg L., Blaschke T. (2005). Adherence to Medication. N. Engl. J. Med..

[207] Zhu Q., Zhu P., Zhang Y., Li J., Ma X., Li N., Wang Q., Xue X., Luo L., Li Z. (2015). Analysis of Social and Genetic Factors Influencing Heterosexual Transmission of HIV within Serodiscordant Couples in the Henan Cohort. PLoS ONE.

[208] Brawner B.M., Kerr J., Castle B.F., Bannon J.A., Bonett S., Stevens R., James R., Bowleg L. (2022). A Systematic Review of Neighborhood-Level Influences on HIV Vulnerability. AIDS Behav..

[209] Nachega J.B., Marconi V.C., van Zyl G.U., Gardner E.M., Preiser W., Hong S.Y., Mills E.J., Gross R. (2011). HIV Treatment Adherence, Drug Resistance, Virologic Failure: Evolving Concepts. Infect. Disord.—Drug Targets.

[210] Maggiolo F., Ravasio L., Ripamonti D., Gregis G., Quinzan G., Arici C., Airoldi M., Suter F. (2005). Similar Adherence Rates Favor Different Virologic Outcomes for Patients Treated with Nonnucleoside Analogues or Protease Inhibitors. Clin. Infect. Dis..

[211] Martin M., Del Cacho E., Codina C., Tuset M., De Lazzari E., Mallolas J., Miró J.-M., Gatell J.M., Ribas J. (2008). Relationship between Adherence Level, Type of the Antiretroviral Regimen, and Plasma HIV Type 1 RNA Viral Load: A Prospective Cohort Study. AIDS Res. Hum. Retroviruses.

[212] Sethi A.K., Celentano D.D., Gange S.J., Moore R.D., Gallant J.E. (2003). Association between Adherence to Antiretroviral Therapy and Human Immunodeficiency Virus Drug Resistance. Clin. Infect. Dis..

[213] Weidle P.J., Nesheim S. (2010). HIV Drug Resistance and Mother-to-Child Transmission of HIV. Clin. Perinatol..

[214] Mirochnick M., Thomas T., Capparelli E., Zeh C., Holland D., Masaba R., Odhiambo P., Fowler M.G., Weidle P.J., Thigpen M.C. (2009). Antiretroviral Concentrations in Breast-Feeding Infants of Mothers Receiving Highly Active Antiretroviral Therapy. Antimicrob. Agents Chemother..

[215] Arrivé E., Newell M.-L., Ekouevi D.K., Chaix M.-L., Thiebaut R., Masquelier B., Leroy V., Van de Perre P., Rouzioux C., Dabis F. (2007). Prevalence of Resistance to Nevirapine in Mothers and Children after Single-Dose Exposure to Prevent Vertical Transmission of HIV-1: A Meta-Analysis. Int. J. Epidemiol..

[216] Pennings P.S. (2012). Standing Genetic Variation and the Evolution of Drug Resistance in HIV. PLoS Comput. Biol..

[217] Hunt G.M., Ledwaba J., Salimo A., Kalimashe M., Dinh T.-H., Jackson D., Sherman G., Puren A., Ngandu N.K., Lombard C. (2019). Prevalence of HIV-1 Drug Resistance amongst Newly Diagnosed HIV-Infected Infants Age 4–8 Weeks, Enrolled in Three Nationally Representative PMTCT Effectiveness Surveys, South Africa: 2010, 2011–12 and 2012–13. BMC Infect. Dis..

[218] Boyce C.L., Sils T., Ko D., Wong-on-Wing A., Beck I.A., Styrchak S.M., DeMarrais P., Tierney C., Stranix-Chibanda L., Flynn P.M. (2022). Maternal Human Immunodeficiency Virus (HIV) Drug Resistance Is Associated with Vertical Transmission and Is Prevalent in Infected Infants. Clin. Infect. Dis..

[219] Castilla J., del Romero J., Hernando V., Marincovich B., García S., Rodríguez C. (2005). Effectiveness of Highly Active Antiretroviral Therapy in Reducing Heterosexual Transmission of HIV. JAIDS J. Acquir. Immune Defic. Syndr..

[220] Tetteh R.A., Yankey B.A., Nartey E.T., Lartey M., Leufkens H.G.M., Dodoo A.N.O. (2017). Pre-Exposure Prophylaxis for HIV Prevention: Safety Concerns. Drug Saf..

[221] Kirby T., Thornber-Dunwell M. (2014). Uptake of PrEP for HIV Slow among MSM. Lancet.

[222] Molina J.-M., Capitant C., Spire B., Pialoux G., Cotte L., Charreau I., Tremblay C., Le Gall J.-M., Cua E., Pasquet A. (2015). On-Demand Preexposure Prophylaxis in Men at High Risk for HIV-1 Infection. N. Engl. J. Med..

[223] Mortlock R., Smith V., Nesci I., Bertoldi A., Ho A., El Mekkawi Z., Kakuzada L., Williams K., Pont L., De Rubis G. (2023). A Comparative Evaluation of Propranolol Pharmacokinetics in Obese versus Ideal Weight Individuals: A Blueprint towards a Personalised Medicine. Chem. Biol. Interact..

[224] Gibas K.M., van den Berg P., Powell V.E., Krakower D.S. (2019). Drug Resistance During HIV Pre-Exposure Prophylaxis. Drugs.

[225] Günthard H.F., Saag M.S., Benson C.A., del Rio C., Eron J.J., Gallant J.E., Hoy J.F., Mugavero M.J., Sax P.E., Thompson M.A. (2016). Antiretroviral Drugs for Treatment and Prevention of HIV Infection in Adults. JAMA.

[226] Christopoulos K.A., Grochowski J., Mayorga-Munoz F., Hickey M.D., Imbert E., Szumowski J.D., Dilworth S., Oskarsson J., Shiels M., Havlir D. (2023). First Demonstration Project of Long-Acting Injectable Antiretroviral Therapy for Persons with and without Detectable Human Immunodeficiency Virus (HIV) Viremia in an Urban HIV Clinic. Clin. Infect. Dis..

[227] Edwards G.G., Miyashita-Ochoa A., Castillo E.G., Goodman-Meza D., Kalofonos I., Landovitz R.J., Leibowitz A.A., Pulsipher C., El Sayed E., Shoptaw S. (2023). Long-Acting Injectable Therapy for People with HIV: Looking Ahead with Lessons from Psychiatry and Addiction Medicine. AIDS Behav..

[228] Ullah Nayan M., Sillman B., Hasan M., Deodhar S., Das S., Sultana A., Thai Hoang Le N., Soriano V., Edagwa B., Gendelman H.E. (2023). Advances in Long-Acting Slow Effective Release Antiretroviral Therapies for Treatment and Prevention of HIV Infection. Adv. Drug Deliv. Rev..

[229] Cunha R.F., Simões S., Carvalheiro M., Pereira J.M.A., Costa Q., Ascenso A. (2021). Novel Antiretroviral Therapeutic Strategies for HIV. Molecules.

[230] Kirtane A.R., Abouzid O., Minahan D., Bensel T., Hill A.L., Selinger C., Bershteyn A., Craig M., Mo S.S., Mazdiyasni H. (2018). Development of an Oral Once-Weekly Drug Delivery System for HIV Antiretroviral Therapy. Nat. Commun..

[231] Oti V.B. (2020). Nanoparticles and Its Implications in HIV/AIDS Therapy. Curr. Drug Discov. Technol..

[232] Rajoli R.K.R., Flexner C., Chiong J., Owen A., Donnelly R.F., Larrañeta E., Siccardi M. (2019). Modelling the Intradermal Delivery of Microneedle Array Patches for Long-Acting Antiretrovirals Using PBPK. Eur. J. Pharm. Biopharm..

[233] Baeten J.M., Hendrix C.W., Hillier S.L. (2020). Topical Microbicides in HIV Prevention: State of the Promise. Annu. Rev. Med..

[234] Walsh S.R., Seaman M.S. (2021). Broadly Neutralizing Antibodies for HIV-1 Prevention. Front. Immunol..

[235] Griffith S.A., McCoy L.E. (2021). To BnAb or Not to BnAb: Defining Broadly Neutralising Antibodies Against HIV-1. Front. Immunol..

